# Harnessing TAGAP to improve immunotherapy for lung squamous carcinoma treatment by targeting c-Rel in CD4+ T cells

**DOI:** 10.1007/s00262-025-03960-1

**Published:** 2025-02-25

**Authors:** Peian Cai, Haibo Sun, Tongmeng Jiang, Huawei Li, Dejing Huang, Xiaopei Hao, Wei Wang, Wenqun Xing, Guanghui Liang

**Affiliations:** 1 Department of Thoracic Surgery, The Affiliated Cancer Hospital of Zhengzhou University & Henan Cancer Hospital, Zhengzhou, 450008 China; 2 Key Laboratory of Emergency and Trauma, Ministry of Education, Engineering Research Center for Hainan Bio-Smart Materials and Bio-Medical Devices, College of Emergency and Trauma, Hainan Provincial Stem Cell Research Institute, Hainan Medical University, Haikou, 571199 China; 3 Department of Thoracic Surgery, The First Affiliated Hospital of Zhengzhou University, Zhengzhou, 450000 China; 4 Department of Hepatobiliary Surgery, The Affiliated Cancer Hospital of Zhengzhou University & Henan Cancer Hospital, Zhengzhou, 450008 China

**Keywords:** T cell activating Rho GTPase-activating protein (TAGAP), CD4+ T cell, Immunotherapy, Prognosis signature, Lung squamous cell carcinoma (LUSC)

## Abstract

Revealing the immunosenescence, particularly in CD4+ T cell function in lung squamous carcinoma (LUSC) assists in devising individual treatment strategies. This study identifies differentially expressed genes (DEGs) between ROS1 mutated (*ROS1*^MUT^) and wild-type (*ROS1*^WT^) LUSC samples from the TCGA database. Using WGCNA, immune-related DEGs (IRGs) were screened. Prognostic signatures derived from IRGs were used to compare immune infiltration, chemotherapy sensitivity, and immune-phenotyping score (IPS) between high- and low-risk subgroups. Hub gene abundance in different cell clusters was analyzed via Sc-seq. TAGAP overexpression or silencing was employed to assess its impact on cytokines production and differentiation of CD4+ T cells, downstream c-Rel expression, and tumor progression. High-risk subgroups exhibited decreased infiltration of natural killer, follicular helper T, and CD8+ T cells, but increased plasma, CD4+ memory resting T, and macrophage M2 cells. These subgroups were more sensitive to Sunitinib and CTLA4 blockade. TAGAP expression was significantly reduced in LUSC. Overexpressing TAGAP enhanced CD4+ T cells to produce cytokines, promoted differentiation into Th1/Th17 cells, inhibited Treg conversion, and suppressed LUSC cell phenotype in vitro. TAGAP overexpression in CD4+ T cells also inhibited LUSC tumor growth and boosted immune infiltration in vivo. TAGAP’s effects on CD4+ T cells were partly reversed by c-Rel overexpression, highlighting TAGAP's role in rejuvenating CD4+ T cells and exerting anticancer effects by inhibiting c-Rel. This study elucidates the novel therapeutic potential of targeting TAGAP to modulate CD4+ T cell activity in immunotherapy for LUSC.

**Supplementary Information:**

The online version contains supplementary material available at 10.1007/s00262-025-03960-1.

## Introduction

**Fig. 1:**
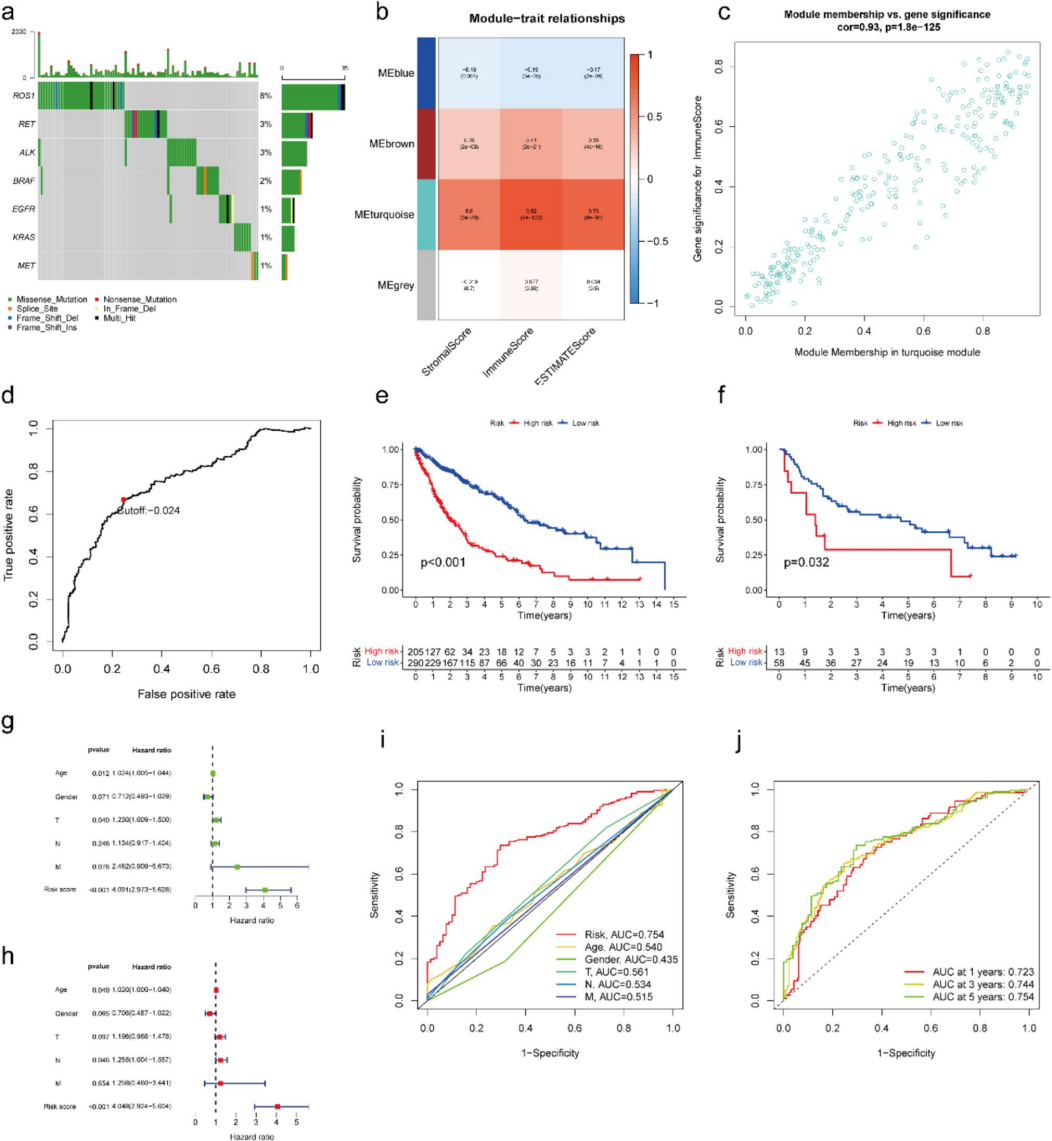
Immune-related prognosis signature construction and efficacy evaluation. **a** Overview of somatic mutations of common therapeutic targets in LUSC of TCGA database. **b** Heatmap of the association between identified modules and TME-related scores of all LUSC samples, including stromal score, immune score and ESTIMATE score. **c** Scatter plot of gene significance for immune score and module membership in the turquoise module. **d** The time-dependent ROC analysis of the prognostic signature to identify the best cut-off value. KM curve of OS in high- and low-risk subgroups of **e** training datasets and **f** validation cohort. **g** Univariate Cox regression and **h** multivariate Cox regression analysis of clinic risk factors in high- and low-risk subgroups of training datasets. **i** Time-dependent ROC analysis of the prognostic signature with other clinic characteristics at 5 years. **j** Time-dependent ROC analysis of the prognostic signature at 1-, 3- and 5-year. LUSC: lung squamous cell carcinoma. TCGA: the Cancer Genome Atlas database. TME: tumor microenvironment. ROC: receiver operating characteristic. KM: Kaplan–Meier. OS: overall survival

As the leading cause of cancer-related deaths, lung cancer is composed of approximately 85% non-small cell lung cancer (NSCLC) which is mainly composed of lung squamous cell carcinoma (LUSC) and lung adenocarcinoma (LUAD) [1, 2]. As the emerging discoveries of molecular targets, the overall survival (OS) of NSCLC increased gradually during the recent year [3] and the overall response rate of NSCLC could be up to 55% [4]. However, as reported, the targeted therapy mainly benefited the non-LUSC and did not bring a satisfactory prognosis to LUSC [5–7]. Hence, as the LUSC occupies 30% of NSCLC, limited treatment strategies for LUSC led to approximately 400,000 deaths every year worldwide [8]. Therefore, a comprehensive understanding of the pathogenic mechanism of LUSC might provide effective targets for the treatment of LUSC. ROS1 is located on the long arm of human chromosome 6, and its full name is c-ros sarcoma oncogene receptor tyrosine kinase (ROS proto-oncogene 1, receptor tyrosine kinase). *ROS1*, along with epidermal growth factor receptor (*EGFR*) and anaplastic lymphoma kinase receptor tyrosine kinase (*ALK*) mutation, are clear driver genes for NSCLC [9]. In addition, ROS1 was found to have a higher mutation rate in LUSC compared with EGFR, ALK and other common mutation genes (Fig. 1a). Therefore, unveiling the mechanisms downstream of ROS1 in LUSC would better construct therapeutics for certain patients.

Increasing evidence suggests the heterogeneity of tumor immune microenvironment (TIME) is a crucial factor affecting tumor development and prognosis, including LUSC [10]. TIME is mainly composed of tumor-infiltrating immune cells (like monocytes, neutrophils, lymphocytes, and tumor-associated macrophages), cancer-associated stromal cells (including endothelial cells, cancer-associated fibroblasts, lipocytes) and neoplastic cells [11, 12]. Besides, specific immune-infiltrating cells have been identified as a practical indicator for distinguishing the clinical stage and outcome of some cancers [13–15]. Immunosenescence, a hallmark of aging, plays a crucial role in TIME [16]. This age-related decline in immune competence is characterized by several key features: a diminished capacity to mount effective responses to novel antigens, a persistent state of low-grade chronic inflammation, the accumulation of memory T cells, and a concomitant reduction in the naïve T cell pool. These factors collectively contribute to widespread T cell dysfunction within the TIME, particularly abnormal of cytokines production and differentiation of CD4+ T cells and impaired cytotoxicity of CD8+ T cells [17]. Many researches have been focused on CD8+ T cell function, especially on programmed death receiver-1 (PD-1) and PD-1 ligand-1 (PD-L1). However, the presence of PD-1 and PD-L1 could make CD8+ T cells inactivate and unable to recognize tumor cells, leading to tumor cell escape [18]. Since 2015, Anti-PD1/PD-L1 immunotherapy has become the standard treatment for stage III-IV NSCLC patients with satisfactory expressions of PD1/PD-L1 [19]. However, due to its relatively low response rates and susceptibility to resistance, anti-PD1/PD-L1 immunotherapy is only beneficial to a small subset of patients and does not produce a lasting response for the majority of patients [20, 21]. Cytotoxic CD4+ T cells could produce massive cytokines to enhance immunotherapy responses in cancer [22]. Thus, exploring the function of CD4+ T cells in the immunosenescence of LUSC may contribute to the development of comprehensive strategies or the fire of new drugs.

TAGAP is a T cell activating Rho GTPase-activating protein widely expressed in natural killer (NK) cells, dendritic cells and B/T lymphocytes and is mainly responsible for T cell activation and cytoskeleton arrangement. As reported, TAGAP is widely involved in some autoimmune diseases and cancer. In rheumatoid arthritis, overexpressed TAGAP could disrupt the balance between Th17 and regulatory T (Treg) cells transformation, promoting Th17 cell differentiation, releasing more inflammatory factors, and exacerbating the progression of rheumatoid arthritis [23]. Moreover, TAGAP in myeloid cells is necessary for proinflammatory cytokine production and anti-fungal signaling activation [24]. More importantly, TAGAP expression was decreased in LUAD and overexpressed TAGAP could limit the tumor progress, thus increasing the toxicity and immune infiltration ability of CD4+ T cells [25]. Conventional CD4+ T cells exhibit enhanced differentiation and polarization toward Th1 and Th1/17 states, leading to the overproduction of cytokines associated with autoimmunity [26]. This phenomenon may be impaired in cancer and immunosenescence situations, which is related to the dysfunction of NF-κB pathway molecules such as c-Rel [27]. However, in LUSC, there is currently little exploration about whether TAGAP can effectively inhibit tumor growth or its molecular mechanisms.

In this work, we identified the differentially expressed genes (DEGs) between *ROS1* mutated (*ROS1*^MUT^) and wild-type (*ROS1*^WT^) LUSC profiles, screened the immune-related DEGs (IRGs) via weighted correlation network analysis (WGCNA), then constructed a reliable immune-related prognostic signature. In the immune-related prognostic signature, TAGAP, as a hub gene, was significantly down-regulated in LUSC samples verified by immunohistochemical (IHC) assay. And TAGAP had a more abundant expression on CD4+ T cells examined by single-cell sequencing (sc-Seq). Rasing TAGAP could rejuvenate the toxicity of CD4+ T cells and inhibit the tumor progress in vitro and in vivo, and vice versa. In terms of mechanism, TAGAP rejuvenized the differentiation of CD4+ T cells toward Th1/17 cells and inhibited their transformation into Treg cells by decreasing the c-Rel expression. The above results suggested that TAGAP would be a potential target for LUSC's future individual clinic strategies.

## Materials and methods

### Data source, preparation and differentially expressed genes (DEGs) analysis

In the present study, 495 LUSC transcriptome, clinical information, as well as VarScan 2-based somatic mutation profiles were harvested from the TCGA database (https://portal.gdc.cancer.gov/). And the LUSC sample from TCGA was set as the training cohort. And 71 LUSC profiles with corresponding prognosis information from GSE19188 of GEO database (http://www.ncbi.nlm.nih.gov/geo/) were set as the validation dataset [28]. The demographic and clinical characteristics of TCGA-LUSC cohort are displayed in Table 1. Besides, to avoid the occurrence of false positive genes in individual samples and also to prevent the erroneous deletion of genes with low expression but significant importance, the genes were removed with the criteria that could not be detected in > 10% of the samples both from training and validation cohorts [29, 30]. And the study was conducted under the TCGA publication guidelines (http://cancergenome.nih.gov/publications/publicationguidelines). The DEGs between *ROS1*^(wt)^ (n = 460) and *ROS1*^(mut)^ (n = 44) were screened via the “limma” package with the threshold of *P* value < 0.05 and |log_2_foldchange (FC)|> 0.5 [31]. The enrichment analyses of the Kyoto Encyclopedia of Genes and Genomes (KEGG) and Gene Ontology (GO) were conducted by “clusterProfiler” algorithm [32]. And its* P* value < 0.05 was thought to be significant.

**Table 1:** Demographic and clinical characteristics of the TCGA-LUSC sample

Characteristics	*ROS1*^(mut)^	*ROS1*^(wt)^
	*n* = 44	*n* = 460
Gender		
Male	31 (70.5%)	342 (74.3%)
Female	13 (29.5%)	118 (25.7%)
Age (Years)		
≥ 65	31 (70.5%)	294 (63.9%)
< 65	13 (29.5%)	157 (34.1%)
Unknown		9 (2)
Smoking		
Yes	38 (86.4%)	389 (84.6%)
Unknown	6 (13.6%)	71 (15.4%)
Location		
Central	12 (27.3%)	135 (29.3%)
Peripheral	8 (18.2%)	85 (18.5%)
unknown	24 (54.5%)	240 (52.2%)
TNM staging		
I	25 (56.8%)	188 (40.9%)
II	11 (25.0%)	132 (28.7%)
III	7 (15.9%)	70 (15.2%)
IV	1 (2.3%)	70 (15.2%)

### Screening the IRGs from DEGs

Weighted gene co-expression network analysis (WGCNA) facilitates the identification of clusters of genes (modules) exhibiting correlated expression patterns and enables the investigation of associations between these modules and phenotypic traits [33]. In the present study, in order to screen the IRGs, the WGCNA algorithm was utilized to identify the gene sets related to the immune infiltration traits among the DEGs. Firstly, the immune score, stromal score and ESTIMATE score of every LUSC sample were evaluated by ESTIMATE via ‘estimate’ package [34]. Following the application of the ESTIMATE algorithm, stromal, immune, and ESTIMATE scores were derived for each LUSC sample. Leveraging these scores as phenotypic data for the LUSC cohort, and utilizing DEGs as the gene expression profiles, we employed WGCNA, as previously described [35], to identify a gene module associated with immune infiltration. A topological overlap matrix was transformed from an adjacency matrix when β = 9 as a result of the connectivity between genes in the gene network meeting a scale-free network distribution. The module associated with the ESTIMATE score > 0.6 and the *P* value < 0.05 was thought to be significant. And the genes that made up the most significant module were identified as the IRGs and were utilized for the immune-related prognosis signature construction.

### Identification and construction for the immune-related prognosis signature

For the construction of the prognostic signature, the IRGs would be paired (IRGsP) firstly, so as to eliminate the influence of measurement error between different samples [36]. If the former gene expression was higher than the latter, the pair was marked as 1, otherwise, it was marked as 0. Next, the IRGsP with uneven distribution (MAD = 0) and low variation (IRGsP score 0/1 in less than 20% samples) would be removed and the remaining IRGsP performed a univariate Cox proportional hazards regression analysis in which the statistically significant IRGsP were further performed the least absolute shrinkage and selection operator (Lasso)-Cox proportional hazards regression with 1000 simulations by the “glmnet” package [37]. Subsequently, the immune-related prognosis signature was constructed based on a final 26 IRGsP signature. And the best cut-off value of the signature was determined by the time-dependent receiver operating characteristics (ROC) for 3 years via the “survival ROC” package [38]. The sample in training or validation cohort was separated into high-/low-risk subgroups based on the immune-related prognosis signature. Finally, in order to evaluate the independent prediction efficiency of signature, a prognostic analysis was performed by log-rank test between the high-/low-risk subgroups to compare the OS difference. In addition, the signature combined with traditionally clinical risk factors of age, gender and TNM staging was conducted univariate and multivariate Cox analyses to further evaluate the independent influence of the immune-related prognosis signature. The ROC curve was utilized for evaluation of the sensitivity and specificity of the signature at 1-, 3-, and 5-year, as well as the above clinical risk factors. The reliability and superiority of immune-related prognosis signature were assessed by area under the curve (AUC) value.

### Comparison of immune infiltration level between high-/low-risk subgroups

CIBERSORT is an algorithm which is developed to elucidate infiltration levels of 22 immune cells (including natural killer cells, plasma cells, naïve and memory B cells, seven T-cell types and myeloid subsets) in bulk tumor based on their gene expression profiles [39]. In order to further explore whether there are differences in immune infiltration cell in tumor tissues between high-risk and low-risk populations, the “CIBERSORT” algorithm was used to elucidate the immune cell infiltration atlas for each subgroup and the Wilcoxon rank-sum test was performed to compare the statistical differences with the threshold of *P* < 0.05.

### Comparison of multiple therapeutic sensitivities between high-/low-risk subgroups

Comparing the sensitivities of multiple therapeutic strategies between high-/low-risk subgroups would help provide more personalized treatment for this population and improve their survival. To compare the sensitivities of chemotherapy and targeted inhibitors (TIs) between the different risk subgroups, the concentrations of 50% reduction growth (IC50) about different chemotherapy and TIs were calculated with the R package “pRRophetic” [40]. And we further screened the potential chemotherapy drugs for LUSC targeting the identified hub genes. Firstly, the gene expressions, as well as the response to various chemotherapy profiles were harvested from CellMiner™ database (https://discover.nci.nih.gov/cellminer/) [41]. The drugs, approved by FDA for clinical treatment, were included to perform the correlation test between gene expressions and IC50. The immunophenotypic score (IPS) is a perfect predictor of anti-PD-1 and anti-CTLA-4 responses and a higher IPS score is associated with superior response to immunotherapy [42]. The IPS scores of every LUSC samples included in the present study were harvested from The Cancer Immunome Atlas database (TCIA, https://tcia.at/) and compared between high-/low-risk subgroups. Unless otherwise specified, the above analyses were all conducted via Wilcoxon rank-sum test with the threshold of *P* < 0.05.

### Nomogram construction and evaluation

To predict the OS more personalized and accurately, a predictive nomogram was constructed based on the prognosis signature, as well as other clinical characters including Gender, Age, and TNM stage distinguished from multivariate Cox regression analysis via the “rms” package [43]. The function of the prognosis signature in the nomogram is to calculate the risk score of a sample based on its 26 gene pairs and their coefficients, and then classify the sample into high-risk or low-risk groups. Furthermore, combined with other clinical characters, the nomogram could more accurately predict its survival probability within 1-,3- 5-years.

Besides, calibration and discrimination are the most common methods for assessing the performance of nomograms [44]. The calibration curve evaluating the OS probability at different years was generated via “rms” package. The 45° line represented the best predictive values and the closer the nomogram predicted probabilities curve is to 45°, the better the performance of nomogram. Besides, discrimination performance of the nomogram was determined by Harrell’s concordance index (C-index) by a bootstrap approach with 1000 resamples to calculate a robust C-index. The C-index value ranged from 0.5 to 1, suggesting a better capacity to correctly distinguish the outcome via the nomogram [45]. Lastly, the C-index and time-dependent ROC were performed to compare the predictive performance between constructed nomogram and traditional prognostic factors.

### Screening and validation of hub genes

To further screen the hub IRGs of LUSC, the IRGs were performed PPI analysis by the STRING database (https://www.string-db.org) [46]. Next, the genes, whose nodes number ≥ 5, significantly expressed between LUSC and normal lung tissues, were identified as the hub gene. TAGAP protein expression in normal lung and LUSC tissue was detected in the database of Human Pathology Atlas (https://www.proteinatlas.org) where the database stores IHC slices of various proteins in different tumors and corresponding normal groups, and quantifies their expression [47].

### Single-cell (sc)-Seq analysis

LUSC sc-Seq profile from GSE127465 (GSM3635285, GSM3635278) of GEO database was utilized to explore which subpopulation in LUSC preferentially expressed TAGAP. In short, we integrated the two profiles (GSM3635285 and GSM3635278) and used the "ComBat" algorithm to eliminate batch effects. The sc-Seq analysis was conducted via the “Seurat” package [48]. Cells with less than 50 genes or more than 5% mitochondrial genes, and clusters with cell numbers less than 3 would be excluded in order to avoid the interference of some false positive genes, low-quality cells and non-representative clusters [49]. Based on the top 2000 highly variable genes, the principal component analysis (PCA) was conducted to extracted the principal components (PC) based on the optimal number from Scree plot result [50]. T-distributed stochastic neighbor embedding (t-SNE) was performed for unbiased visualization and unsupervised clustering. And the expression of target gene among different was detected via “FindAllMarkers” function. The annotation of cell subpopulations was conducted via the “SingleR” package.

### Cell culture, sorting and treatment

LUSC cell lines: NCI-H226 and SK-MES-1, human peripheral blood mononuclear cells (hPBMCs) from healthy individuals, were all purchased from American Type Culture Collection (ATCC, USA). The LUSC cell were culture with RPMI 1640 medium (Gibco, USA) [51]; while, the hPBMCs were cultured in DMEM medium (Gibco, USA) [52], both of medium containing 10% FBS (HyClone, USA).

Next, the CD4 Positive Isolation Kit (Miltenyi Biotec, Germany) was utilized for CD4+ T cell sorting from hPBMCs according to the operating manual with a magnetic sorter system. Briefly, the PBMCs suspended in the buffer were incubated with CD4+ T Cell Biotin-Antibody Cocktail for 5 min at 4 °C. Next, the above mixture was added to CD4+ T Cell MicroBead Cocktail and incubated for 10 min at 4 °C. Subsequently, the mixture was transformed into the column in the magnetic field of MidiMACS Separator (Miltenyi Biotec, Germany). The enriched CD4+ T cells were collected from the flow-through solution containing unlabeled cells. And the cell purity was assessed by flow cytometer. Lastly, the CD4+ T cells were activated with anti-CD28 (2 mg/mL) and anti-CD3 (4 mg/mL) monoclonal antibodies (eBioscience, USA) [53].

TAGAP, c-Rel overexpression plasmid (named as TAGAP (ov), c-Rel (ov)) containing the TAGAP or c-Rel full-length open reading fragment and corresponding control plasmid (NC (ov)), small interfering RNA (siRNA) target the sequence: GCAAGCCCAGCCGAGAAATTA for silencing TAGAP expression (TAGAP (si)) and corresponding control siRNA (NC (si)), were all obtain from GenePharma Corporation (China). All the transfection assay were conducted with Lipofectamine 3000 (Invitrogen, USA) according to the operating manual.

### Western blot assay

To examine the efficiency of CD4+ T cells transfected with TAGAP (ov), c-Rel (ov) and TAGAP (si), we performed a western blot assay on the above-treated cells. The total protein sample were harvested via RIPA solution (Beyotime, China) supplemented with 1 mM PMSF (Beyotime, China) and the concentration was measured with BCA kit (Beyotime, China). Briefly, a total of 50 µg protein sample was separated with 10%SDS-PAGE and transferred onto a PVDF membrane (Roche, Switzerland). Next, the membrane was incubated with primary antibodies including β-actin (20536-1-AP, dilution: 1: 5,000; Proteintech, China), TAGAP (ab187664, dilution: 1: 1,000; abcam, USA), c-Rel (ab133251, dilution: 1: 1,000; abcam, USA) at 4 °C overnight, followed by incubation with HRP-conjugated secondary antibody (ZB-2306, dilution: 1: 20,000; ZSGB-BIO, China) at room temperature for 1 h. Finally, the membranes were visualized with ECL kit (Beyotime, China) via ChemiDoc MP System (Bio-Rad. USA) and quantified with ImageJ.

### Cell cytotoxicity assay

To observe TAGAP effect on the toxicity of CD4+ T to LUSC cells, we co-cultured TAGAP overexpressing or silencing CD4+ T cells with LUSC cell. Firstly, the LUSC cells were seeded into the 96-well plate at a density of 5000/well for 24 h. And then, CD4+ T cells with TAGAP overexpression or silence were added to the LUSC cell well at 1:2, 1:3, and 1:5 ratios for another 24 h. Finally, the NCI-H226 or SK-MES-1 cellular cytotoxicity level was detected with LDH cytotoxicity assay kit (Beyotime, China) according to the operating manual [25]. Briefly, remove the culture medium of NCI-H226 and SK-MES-1 cells and wash the cells three times with PBS. Then, the LDH release reagent was supplied to the cells and incubated at 37 °C for 1 h. Next, the supernatant was collected and supplied with an LDH detection working solution, followed by being incubated at room temperature for 30 min. Finally, the OD value was detected at 490 nm and the LDH activities of each sample were measured via the standard curve. The data were normalized by calculating the relative multiple of LDH activity in each group compared with that in the control group.

### Apoptosis assay

To observe the apoptosis level of LUSC cells co-cultured with CD4+ T cells of overexpression or silencing TAGAP, the LUSC cells were firstly co-cultured with CD4+ T cell at the ratio of 1:3 for 24 h. Next, the CD4+ T cell would be removed through removing the culture medium [54]. Subsequently, the NCI-H226 and SK-MES-1 cells were collected and incubated with propidium iodide (PI) and annexin V solution in the dark at room temperature for 10 min. After centrifugation at 500×*g* for 5 min to remove the staining solution, the cells were resuspended in PBS and tested with the FACSCanto II system (BD Pharmingen, USA). The data were analyzed with FlowJo software.

### Scratch wound experiment

To observe the migration level of LUSC cells co-cultured with CD4+ T cells, the scratch wound assay was conducted. Briefly, LUSC cells were inoculated into a 6-well plate and cultured until cell confluence reached approximately 90%. Next, a 200 μl pipette was utilized to scratch on the monolayers. After the cell debris was removed by PBS, the CD4+ T cells with the overexpression or silencing TAGAP were added into the well at the ratio of 1:3 for co-culture for another 24 h. Finally, the scrape lines were captured at 24 h and the migration rate was calculated with ImageJ.

### Flow cytometric assay

The flow cytometric assay was utilized to investigate whether TAGAP could affect the activation (marker as: CD25/CD69) and differentiation (Treg cell marker: FOXP3, Th1 cell marker: IFN, Th17 cell marker: IL17A) of CD4+ T cells. The CD4+ T cells with overexpression or silencing TAGAP was treated with anti-CD4-FITC (553,046, Clone: RM4-5, BD, USA), anti-CD25-APC (561,048, Clone: RM4-5, BD, USA), anti-CD69-PE (561,932, Clone: H1.2F3, BD, USA) and anti-FOXP3-PE (561,932, Clone: MF23, BD biosciences, USA) antibodies for 1.5 h before conducting the cytometric assay. While IFNγ and IL17A detections, T cells were pre-stimulated with phorbol 12 myristic acid 13 acetate (PMA), ionomycin and GolgiPlug (Sigma Aldrich, USA) for 4 h to prevent the secretion of cellular cytokines and be beneficial for intracellular staining, before anti-IL17A-PE (561,932, Clone: B27, BD, USA), anti-IFN-γ-PE (562,018, Clone: XMG1.2, BD, USA) as well anti-CD4-FITC (553,046, Clone: RM4-5, BD, USA) staining. And cell single-stained with anti-CD4-FITC was set as the Control group for the above double-stain group. Finally, the stained cells were processed with the FACSCanto II system (BD, USA) and analyzed by FlowJo software.

### Enzyme-linked immunosorbent assay (ELISA)

To investigate whether TAGAP can regulate the cytokine levels of CD4+ T cells, the ELISA assay was conducted to detect the levels of IFNγ, IL17A, and IL10 in CD4+ T cell culture medium with TAGAP overexpression or silence. Briefly, the cell supernatant was obtained by centrifugation at 500×*g* for 5 min. The supernatant was utilized to conduct the assay according to the operating manual of IFNγ, IL17A, and IL10 ELISA kits (R&DSystems, USA). The OD value was detected and the concentrations of IFNγ, IL17A, and IL10 were calculated with the corresponding standard curve according to the instruction manual.

### Experimental animals and treatment

All experimental procedures followed the “Guidelines for the Care and Use of Experimental Animals” and were approved by the Animal Care and Utilization Ethics Committee of Zhengzhou University (No.ZZU-LAC20240322). 32 NSG mice (4–5 weeks, female, Charles River, China) were raised in the Animal Experimental Center of Zhengzhou University. And each mouse was inoculated subcutaneously 100 µl PBS containing 5 × 10^6^ NCI-H226 or SK-MES-1 cells on the right flank. When the tumor volume grew up to 100 mm^3^, 4 groups for each cell line were divided randomly: NC (ov): receiving 1 × 10^7^ CD4+ T cells treated with NC (ov) of TAGAP plasmid, intravenously; TAGAP (ov): receiving 1 × 10^7^ TAGAP overexpression CD4+ T cells, intravenously; NC (si): receiving 1 × 10^7^ CD4+ T cells treated with NC (si) of TAGAP, intravenously; TAGAP (si): receiving 1 × 10^7^ TAGAP silencing CD4+ T cells, intravenously. Subsequently, the tumor size was recorded every 4 days. And the humane endpoints were set as: the max tumor volume was no more than 1687.5 mm^3^ or the length of tumor was no more than 15 mm. During the observation of 28 days, no mice reached the humane endpoint to be euthanized early. And after the observation is completed, all the mice were euthanized via cervical dislocation and the tumors were harvested and measured. The tumor volume was calculated following the formula: Volume s = width^2^ × length/2. The tumor sample was prepared as paraffin sections for subsequent assay or long-term storage.

### Fluorescence staining

Detecting CD4 expression used for evaluating the infiltration level of CD4+ T cells in tumor samples. Briefly, after high-temperature repair with sodium citrate buffer (Boster, China), the paraffin sections were treated with 3% H_2_O_2_ to block endogenous peroxidation activity. And 10% goat serum (Zhongshan Jinqiao, China) was used to block non-specific binding sites. Next, the primary antibody CD4 (ab133616, dilution: 1: 500; Abcam, USA) incubated the slices at 4 °C overnight, followed by incubation with IgG H&L antibody (ab150077, dilution: 1: 1,000; Abcam, USA) at room temperature for 1 h. Finally, the nucleus was stained by DAPI (Solarbio, China) solution at room temperature for 5 min. And the images were captured via a fluorescence microscope (OLYMPUS, Japan).

### Terminal deoxynucleotidyl transferase-mediated dUTP nick-end labeling (TUNEL) assay

The TUNEL assay was used to investigate whether silencing or overexpression of TAGAP in CD4+ T cells could affect the apoptosis level of tumor cells in vivo. First, the tumor sample was fixed in the 4% paraformaldehyde at 4 °C for 24 h. After dehydration using xylene and gradient alcohol solution, paraffin-embedded tissues were prepared and utilized for 3-µm slice making. The TUNEL staining was performed according to the manufacturer’s protocol of TUNEL staining kit (Roche, Netherlands). Briefly, the slices were sequentially dewaxed with xylene and hydrated with gradient alcohol. Next, the slices were incubated with DNase I free protease K (20 μg/ml), dissolved in 10 mM Tris–HCL solution, at room temperature for 30 min. Subsequently, add TUNEL working solution onto the slices and incubate the sample in dark for 1 h at 37 °C. In order to reduce the noise of the background, the slices were washed with PBS containing 0.1% Triton X-100 and 5 mg/ml BSA for 3 times, 5 min each time. Finally, the nucleus was stained by a DAPI (Solarbio, China) solution. And the images were captured via a fluorescence microscope (OLYMPUS, Japan).

### Statistical analysis

In this study, unless otherwise stated, all experiments were conducted at least three times, with results expressed as the mean ± standard deviation (SD). Statistical analyses and chart generation were carried out using GraphPad Prism software (version 8.0). For statistical comparisons between two groups, an unpaired t test was employed; while, comparisons among three or more groups were conducted using one-way ANOVA followed by Tukey's multiple comparison test. A *P*-value of less than 0.05 was considered statistically significant.

## Results

### Identification of IRGs among the DEGs between ROS1^mut^ and ROS1^wt^group

Firstly, we explored the mutation situation of *EGFR*, *KARS*, *MET, ALK*, *BRAF*, *RET* and *ROS1* in LUSC. Obviously, *ROS1* mutation frequency was the highest (8%) and most of them were missense mutations (Fig. 1a). There were 796 DEGs with 212 up- and 584 down-regulated genes. Subsequently, the potential biological functions of DEGs were presented by GO and KEGG enrichments. As for biological process (BP), the DEGs were mainly involved in the signaling transduction, immune response, inflammatory response, transcription from DNA/RNA, apoptosis and protein phosphorylation processes (Fig. S1a). And in the cellular component (CC) term, the cytosol, cytoplasm and membrane was the significant term enriched by DEGs (Fig. S1b). In the molecular function (MF) category, the DEGs have mainly participated in the protein, ATP, antigen and receptor binding (Fig. S1c). In the KEGG terms, besides pathways in cancer, cytokine-cytokine receptor interaction was the most significant result (Fig. S1d). These results suggested the DEGs between *ROS1*^*mut*^* and ROS1*^*wt*^ LUSC group were mainly involved in the immune activities via protein binding of cell receptor.

Next, WGCNA was applied to further screen the IRGs among the DEGs. Firstly, the immune infiltration levels of every LUSC sample were evaluated which was assessed by three related factors: stromal, immune, and ESTIMATE scores (Table S1). Next, the DEG profiles, as well as the immune infiltration levels of LUSC samples were utilized for WGCNA. After outlier data exclusion, a soft threshold power of *β* value was set as 9 because the connectivity between genes in the gene network met a scale-free network distribution with the scale-free R^2^ value close to 0.9 (Fig. S2a). A scale-free co-expression network containing 796 DEGs was constructed (Fig. S2b). For the results, the turquoise module was significantly associated with a stromal score (Cor = 0.6), immune score (Cor = 0.82) and ESTIMATE score (0.76) (Fig. 1b). Moreover, a high immune score of gene significance correlated with module membership (Cor = 0.93) had been found in genes in the turquoise module (Fig. 1c). These results suggested that the genes in the turquoise module were suitable for being recognized as IRGs.

### An immune-related prognosis signature construction based on the IRGs

A total of 286 genes in the turquoise module were thought to be the IRGs and were selected for the immune-related prognosis signature construction. In addition, after conducting univariate Cox proportional hazards regression and Lasso Cox proportional hazards regression analyses, a total of 26 IRGsP with their risk coefficients was obtained (Table 2). With the prognosis signature, the sample risk score in the training cohort was evaluated and the best cut-off value (-0.024) was determined based on the 3-year ROC curve (Fig. 1d). Based on the cut-off value, the samples were divided into low- and high-risk subgroups. As expected, the OS in the high-risk subgroup was worse than that in the low-risk subgroup (Fig. 1e). Besides, the same result was also shown in the validation dataset where the outcome in the low-risk subgroup was better than that in the high-risk subgroup which was divided by the prognostic signature (Fig. 1f). Furthermore, the signature was performed the univariate and multivariate Cox regression analyses with clinical risk factor of LUSC (Age, Gender, TNM stages) and the results suggested the prognostic signature was an independent prognostic factor for LUSC (Fig. 1g-h). Besides, the AUC values of the signature were 0.753, which were higher than that of Age, Gender, TNM stages values at 5-year (Fig. 1i). Moreover, the AUC value of 1-, 3- and 5-year OS were 0.723, 0.744 and 0.754 (Fig. 1j). These results suggested that signature had good effectiveness in the prognostic evaluation of LUSC.

**Table 2:** Prognostic signature information with26 IRGsP

Gene	Coef.
ADAMDEC1|GFPT2	−0.04506
AIM2|IL18R1	0.279912
AOAH|AOC3	−0.08013
AOAH|IL2RA	−0.04986
AOAH|SAMD9L	−0.10771
AOAH|SERPINB9	−0.10108
AOAH|SLIT2	−0.00712
CD180|TRAF3IP3	0.145911
CD37|GAS1	−0.15336
CD37|MMP19	−0.09163
CHI3L1|MEST	0.078511
CYTIP|MMP19	−0.15078
DAPP1|TAGAP	−0.17731
DOCK2|IL18R1	0.181694
EGR2|PLAC8	0.03231
EGR2|TSPAN33	0.055216
ERLIN2|SERPINB9	−0.13074
ICAM3|THEMIS	0.143621
IL10RA|MMP19	−0.10313
IL2RG|SELPLG	−0.13703
ITGAL|MMP19	−0.02076
ITGB7|MILR1	−0.21373
KLHL6|TRAF3IP3	0.359815
LSM1|TRIM22	−0.11753
NCF1|P2RY8	−0.06368
REEP6|SERPINB9	−0.12878

### Comparison of immune infiltration, sensitivity of chemotherapy, and IPS differences between low-/high-risk subgroups

**Fig. 2:**
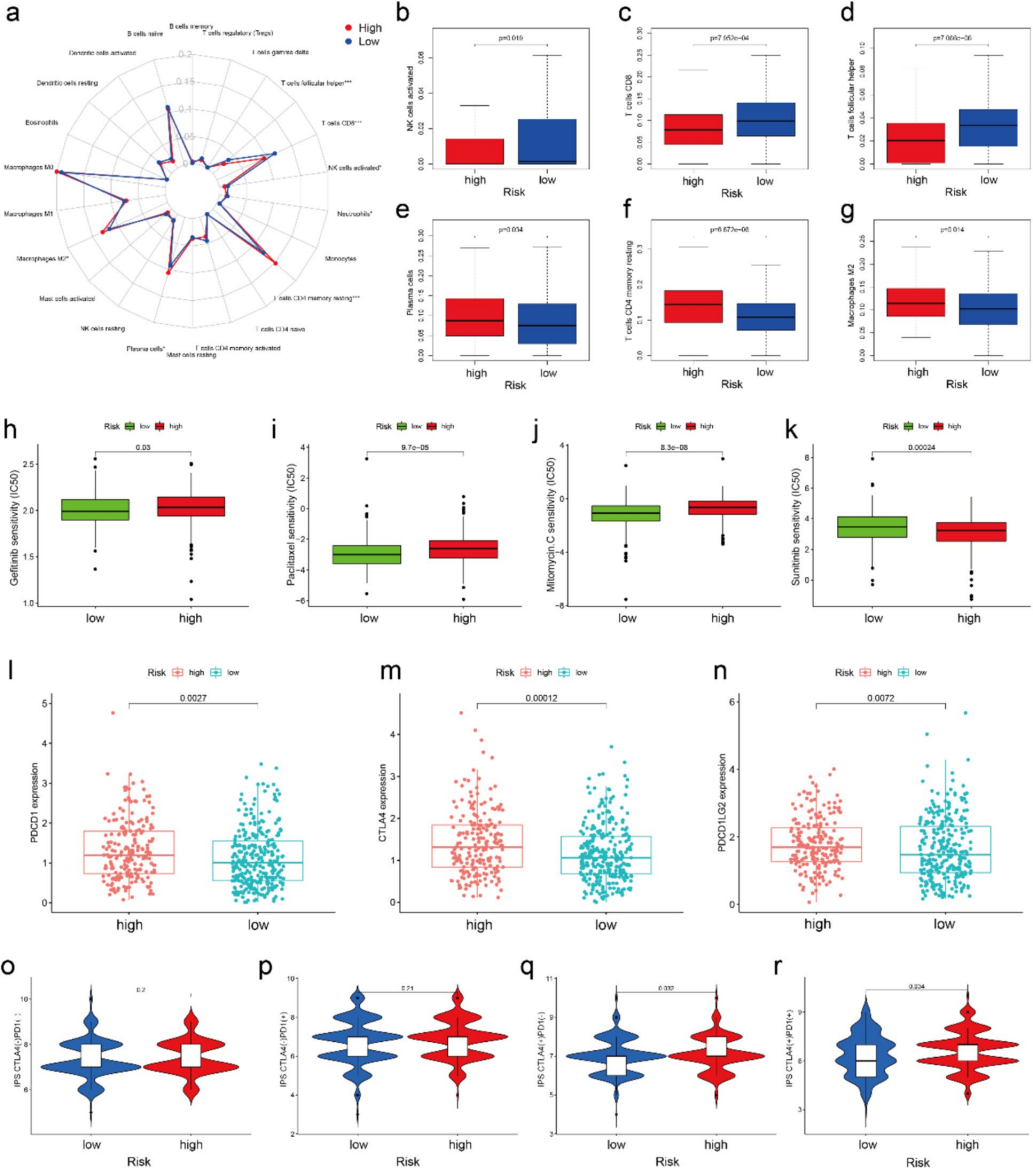
Comparison of immune infiltration, sensitivity of chemotherapy, and IPS differences between high-/low-risk subgroups. **a** The summary of the 22 types of immune cell infiltration levels estimated by “CIBERSORT” between the different risk subgroups. The boxplots for immune infiltration levels of **b** NK cell **c** CD8+ T cell **d** follicular helper T cell **e** plasma **f** CD4+ memory resting T cell and **g** M2 macrophage between the different risk subgroups. The comparison of various chemotherapy sensitivities between the different risk subgroups, including **h** Gefitinib, **i** Paclitaxel, **j** Mitomycin.C, **k** Sunitinib. The comparison of ICIs-related gene expression levels containing **l** PDCD1, **m** CTLA4 and **n** PDCD1LG2 between different risk subgroups. **o–r** The association between IPS and risk subgroups of LUSC with different immunotherapy decisions. **o** CTLA4(−)/PDL1 (−). **p** CTLA4(−)/PDL1 (+). **q** CTLA4(+)/PDL1 (−). **r** CTLA4(−)/PDL1 (+). IPS: immune-phenotyping score. NK: natural killer cell. ICIs: immune checkpoint inhibitors. LUSC: lung squamous cell carcinoma

The gap in infiltration levels in low- and high-risk subgroups are shown in Fig. 2a. The infiltration levels of NK, CD8+ T and follicular helper T cells in the high-risk subgroup were significantly lower compared with their levels in the low-risk subgroup (Fig. 2b-d). However, the infiltration levels of plasma, CD4+ memory resting T and macrophage M2 cells in the low-risk subgroup were significantly lower in comparison with their levels in the high-risk subgroup (Fig. 2e-g). It is worth noting that the IC50 of Gefitinib (Fig. 2h), Paclitaxel (Fig. 2i), and Mitomycin.C (Fig. 2j) were higher in the high-risk subgroup and Sunitinib (Fig. 2k) was lower in the high-risk subgroup. There was no statistically significant difference in IC50 between Cisplatin (Fig. S3a) and Lapatinib (Fig. S3b) in the high-risk and low-risk groups. These results suggested Sunitinib might be a potential chemotherapy drug for the high-risk subgroup of LUSC. Immunotherapy has been proven to benefit some patients. The immune checkpoint inhibitor (ICI) expressions of PDCD1 (Fig. 2l), CTLA4 (Fig. 2m) and PDCD1LG2 (Fig. 2n) were higher in the high-risk subgroup compared with those in the low-risk subgroup. We noticed that a higher IPS was found in the high-risk subgroup than the low-risk subgroup in CTAL4 positive LUSCs samples (Fig. 2o-r). Therefore, anti-CTAL4 therapy might be potentially beneficial for high-risk patients of LUSC.

### Prognosis-related nomogram construction for OS evaluation

**Fig. 3:**
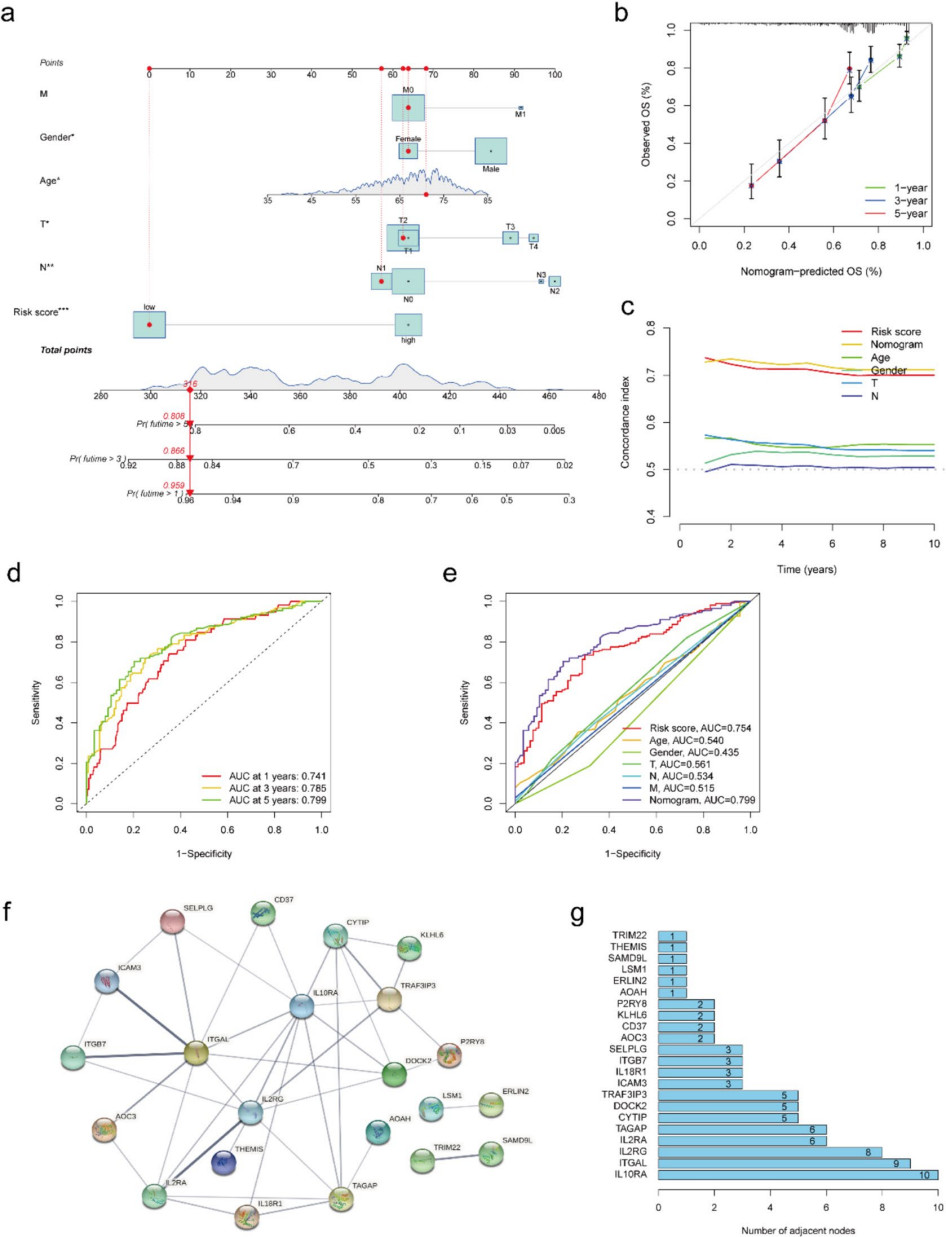
Nomogram construction and hub genes screening. **a** Nomogram for predicting the 1-, 3-, and 5-year OS of LUSC. **b** Calibration curve for evaluating the accuracy and effectiveness of the nomogram at 1-, 3- and 5-years OS. **c** Concordance index of the nomogram, prognosis signature and other risk factors. **d** Time-dependent ROC curve analyses of the nomogram at 1-, 3- and 5-years. **e** Time-dependent ROC curve analyses of the nomogram, prognosis signature and other risk factors at 5 years. **f** The PPI analysis for IRGs (gene without any nodes was excluded). **g** Gene list ranked by gene nodes. LUSC: lung squamous cell carcinoma. OS: overall survival. ROC: receiver operating characteristic. PPI: protein–protein interaction

To predict the prognosis of LUSC patients individually, a nomogram combined with clinical information (Age, Gender and TNM stages) and the prognosis signature was established to evaluate the 1-, 3- and 5-year OS for LUSC patients (Fig. 3a). For example, a LUSC patient (72 years old, Female, T2N1M0) with the total score was 316 and her 1-, 3- and 5-year survival rate was 0.959, 0.866 and 0.808, respectively (Fig. 3a). Moreover, the rational coherence in the predicted model and the experimentally observed model of the 1-, 3- and 5-year outcome were demonstrated by the calibration (Fig. 3b). Furthermore, the predictive accuracy of the nomogram was compared with prognosis signature and clinicopathological factors (age, gender, T stage, and N stage) using the C-index. Our analysis demonstrated that the nomogram exhibited superior performance compared to these individual factors and prognosis signature (Fig. 3c). The AUC values of the time-dependent ROC curve of the constructed nomogram for OS at 1-, 3- and 5-year were 0.741, 0.785, 0.799, respectively (Fig. 3d). As expected, the AUC value of nomogram was also higher than clinicopathological factors (age, gender, T stage, and N stage) and prognosis signature at the 5-year (Fig. 3e). Therefore, the nomogram based on the signature could be more personalized and accurately to predict the OS of LUSC.

### Screening the potential chemotherapeutic drugs

It was beneficial for some subgroups of LUSC to explore potential chemotherapy drugs. To explore the new drugs, the PPI analysis was performed to identify the hub genes among the signature (Fig. 3f). The genes with the nodes number ≥ 5 (IL10RA, ITGAL, IL2RG, IL2RA, TAGAP, CYTIP, DOCK2 and TRAF3IP3) were thought to be the hub genes of the network of signature (Fig. 3g) and were utilized to screen the drugs with the highest correlation. As shown in Fig. S4, TRAF3IP3 and IL2RG had the most correlation with Nelarabine and Chelerythrine. And TAGAP was significantly associated with Imexon. ITGAL had a higher correlation with Chelerythrine, Nelarabine, Hydroxyurea, and Imexon. DOCK2 was significantly associated with Hydroxyurea, Chlorambucil, Nelarabine, Chelerythrine and Uracil mustard. CYTIP was most significantly related to Isotretinoin and Fluphenazine. And the results revealed that the above drugs might be effective for LUSC patients with TRAF3IP3, IL2RG, TAGAP, ITGAL, DOCK2, and CYTIP abnormal expressions.

### Screening the potential molecular mechanism for LUSC

**Fig. 4:**
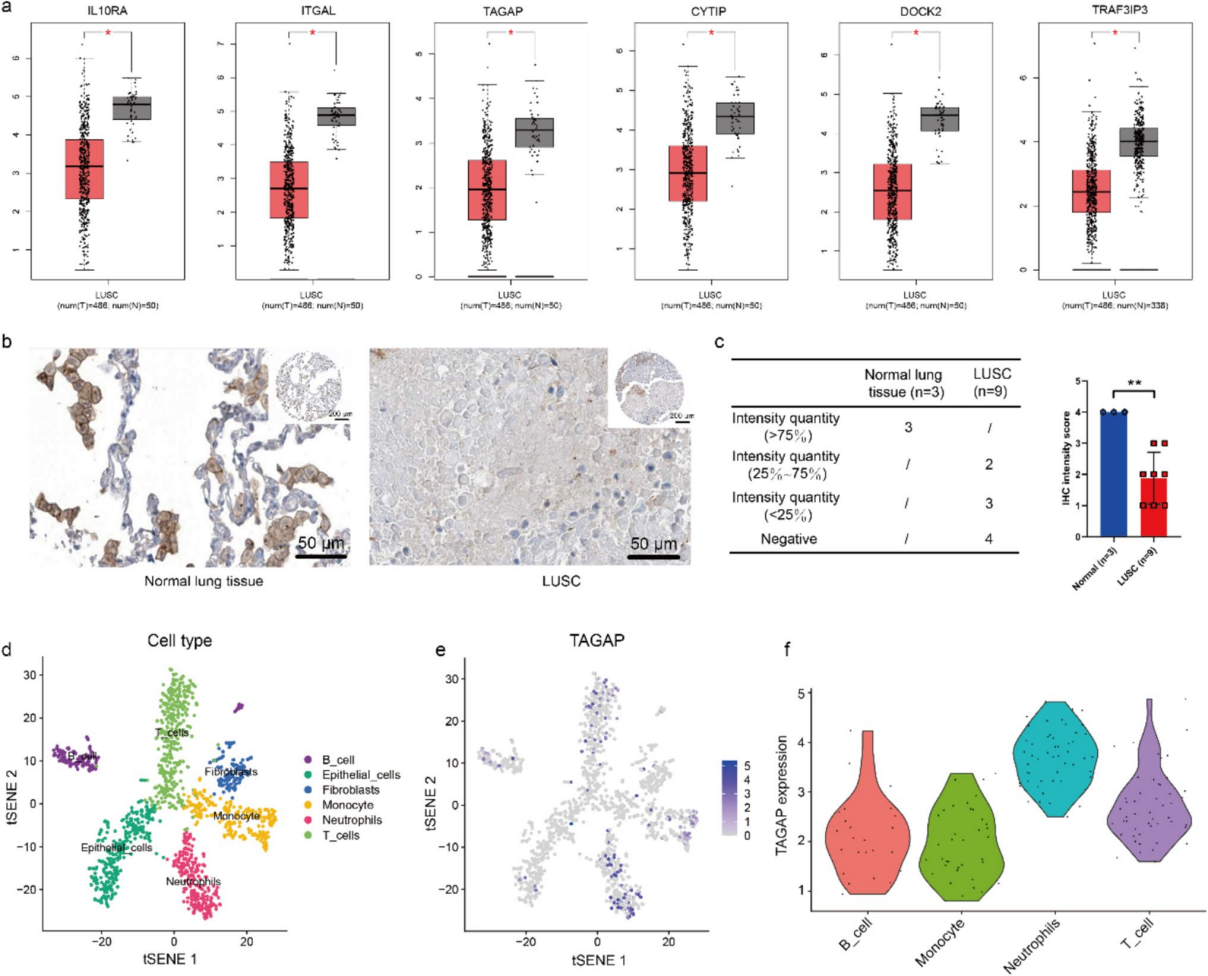
Potential molecular target screening and validation. **a** Difference analysis of hub genes between LUSC and normal samples. **b** The protein expression of TAGAP in LUSC and normal lung tissue via IHC assay (scale bar = 50 µm). **c** Statistical analysis of TAGAP expression in LUSC and normal lung tissue (**P < 0.01). **d** The different cell populations analyzed via sc-seq sequencing. **e** The expression and distribution of TAGAP in different cell populations of LUSC. **f** The expression of TAGAP in the immune cell of LUSC. LUSC: lung squamous cell carcinoma. KM: Kaplan–Meier. OS: overall survival. IHC: immunohistochemistry. sc-Seq: single-cell sequencing

In the hub genes, the expressions of IL10RA, ITGAL, TAGAP, CYTIP, DOCK2, and TRAF3IP3 were significantly down-regulated in LUSC compared with that in paracancerous samples (Fig. 4a). Among them, we paid particular attention to TAGAP, as TAGAP is mainly expressed in immune cells and is responsible for T cell activation and cytoskeleton arrangement. And the IHC assay verified the expression of TAGAP in the LUSC sample (Fig. 4b) in which TAGAP expression was decreased in LUSC in comparison with the normal lung sample (Fig. 4c). By dissecting the sc-seq profiles of LUSC, the Scree plot result suggested 15 as the most rational number of principal components (Fig. S5). A total of 6 cell subsets were detected via tSNE analysis containing B cells, epithelial cells, fibroblasts, monocytes, neutrophils, and T cells (Fig. 4d). Among the immune cells, TAGAP was mainly expressed in the neutrophils and T cells (Fig. 4e-f). Therefore, we investigated the role of TAGAP in CD4+ T cells in the following experiments.

### CD4+ T cells overexpressing TAGAP could inhibit the proliferation and migration of tumor cells

**Fig. 5:**
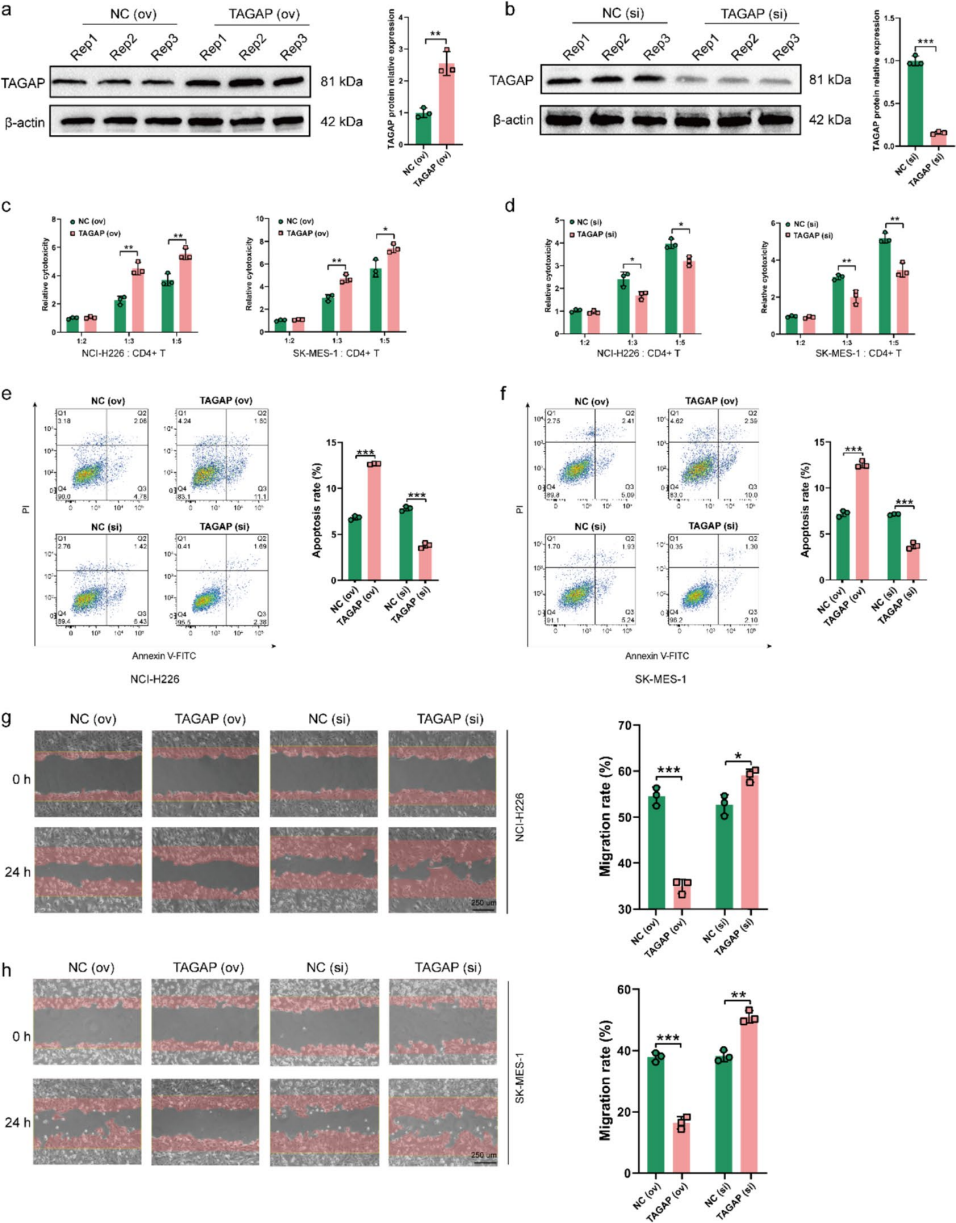
TAGAP of CD4+ T cell affected the development of LUSC cells. The protein expression of TAGAP in CD4+ T cell with **a** TAGAP (ov) plasmid and **b** TAGAP (si) application. The cytotoxicity changes of CD4+ T cells with **c** TAGAP (ov) plasmid and **d** TAGAP (si) application. The **e** NCI-H226 and **f** SK-MES-1cellular apoptosis levels change with TAGAP overexpressing or silencing CD4+ T cell treatment. The **g** NCI-H226 and **h** SK-MES-1 migration levels change with TAGAP overexpressing or silencing CD4+ T cell treatment (scale bar = 250 µm). *P < 0.05, **P < 0.01, ***P < 0.001

After sorting, CD4+ T cell purity was identified via flow cytometry, and the results showed that the purity of CD4+ T cells was 97.3% after sorting (Fig. S6a). The efficiency of overexpression and silencing of TAGAP was confirmed by a western blot assay. The CD4+ T cell significantly overexpressed TAGAP after TAGAP (ov) plasmid treatment (Fig. 5a); while, TAGAP expression was decreased with TAGAP (si) application (Fig. 5b). Next, to investigate whether TAGAP could affect the toxicity of CD4+ T cells, activated CD4+ T cells overexpressing or silencing TAGAP were co-cultured with NCI-H226 or SK-MES-1, respectively, and subjected to LDH assay. The results suggested TAGAP (ov) significantly increased the toxicity when the target-to-effector ratio of the co-culture system was set as 1:3, 1:5 (Fig. 5c). While TAGAP (si) weaken the toxicity of activated CD4+ T cell (Fig. 5d). In addition, in the co-culture system, TAGAP (ov) could increase the apoptosis ratio; while, TAGAP (si) decreased the apoptosis level in both NCI-H226 and SK-MES-1 cells (Fig. 5e-f). Cell migration is a crucial characteristic of tumor cells. We also explored whether TAGAP could affect the migration ability of LUSC cells in the co-culture system. The results revealed that TAGAP (ov) could inhibited the migration ability and TAGAP (si) could strengthen the ability of migration (Fig. 5g-h). The above results indicated that TAGAP of CD4+ T cells might inhibit the proliferation and migration of LUSC cells by regulating the cytotoxicity of CD4+ T cells.

**Fig. 6:**
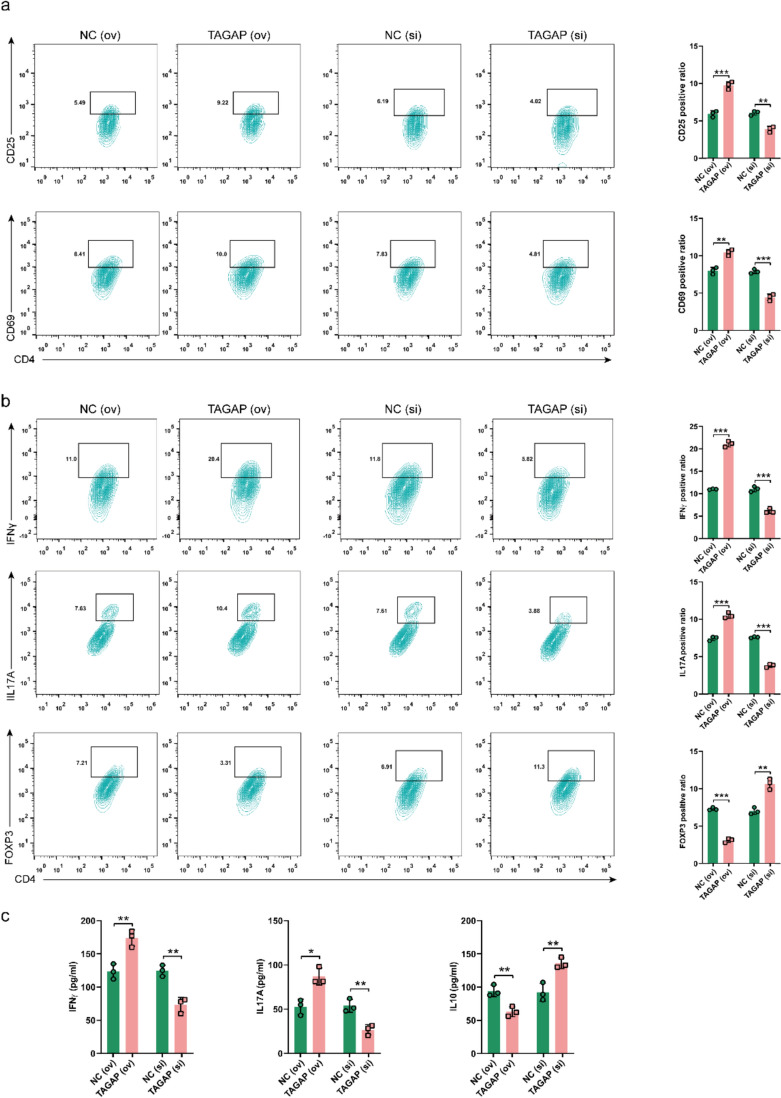
TAGAP regulated the differentiation of CD4+ T cells. **a** CD25 and CD69 expression on CD4+ T cells after TAGAP overexpressing or silencing. **b** The percentages of Th1 (CD4+ and IFN-γ), Th17 (CD4+ and IL17A+), and Treg (CD4+ and FOXP3+) changes after TAGAP overexpressing or silencing in CD4+ T cell. **c** The cytokine levels of IFN-γ, IL17A and IL10 after TAGAP overexpressing or silencing in CD4+ T cell. *P < 0.05, **P < 0.01, ***P < 0.001

The above results encouraged us to investigate whether TAGAP also affects other functions of CD4+ T cells. Fig. S6b displayed the Control and gating strategies used in flow cytometry analysis. And the results revealed TAGAP (ov) significantly increased the activation markers (CD25 and CD69) of CD4+ T cell; while, TAGAP (si) significantly inhibited this phenomenon (Fig. 6a). Moreover, TAGAP (ov) increased the proportions of Th1 (calculated by CD4+ and IFNγ) and Th17 cells (calculated by CD4+ and IL17A), and decreased the Treg cell (calculated by CD4+ and FOXP3) proportion. However, TAGAP (si) inhibited the activation of CD4+ T cells, suppressed their differentiation into Th1 and Th17 cells, and promoted their development into Treg cells (Fig. 6b). In addition, to further investigate whether TAGAP can regulate the cytokines production of CD4+ T cells, the cytokines levels of IFNγ, IL17A and IL10 were detected. The results suggested TAGAP (ov) increased the production of IFNγ and IL17A, and decreased IL10 secretion. And TAGAP (si) generated an opposite result (Fig. 6c). These results suggested TAGAP participated in regulating the activation and differentiation of CD4+ T cell.

### CD4+ T cells overexpressing TAGAP could inhibit tumor development in vivo

**Fig. 7:**
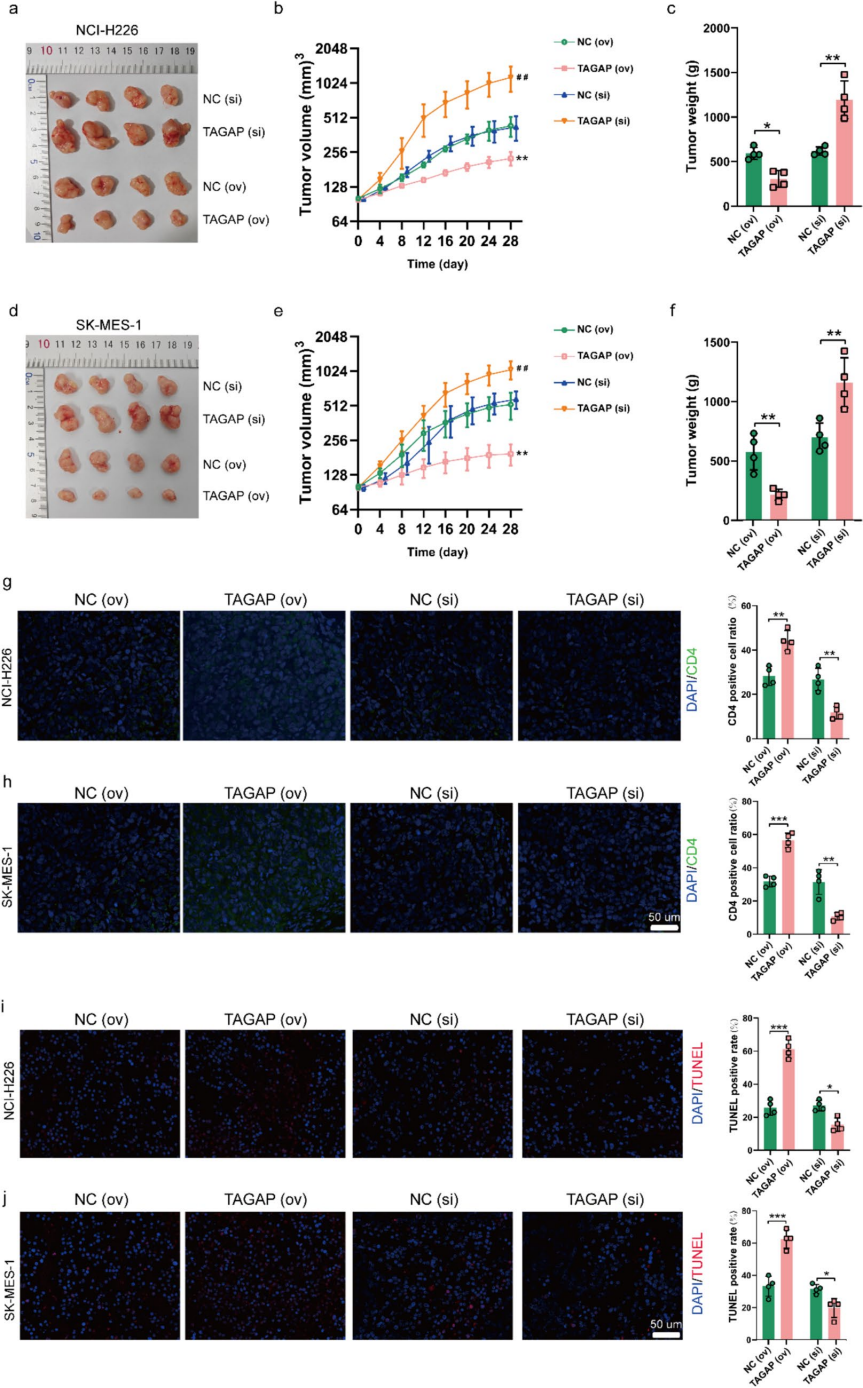
CD4+ T cells with TAGAP overexpression inhibited tumor development in vivo. The represent images for **a** NCI-H226 and **d** SK-MES-1 subcutaneous tumor with CD4+ T cells of TAGAP overexpressing or silencing treatment. The comparison of tumor volume of **b** NCI-H226 and **e** SK-MES-1 with CD4+ T cells of TAGAP overexpressing or silencing treatment. The comparison of tumor weight of **c** NCI-H226 and **f** SK-MES-1with CD4+ T cells of TAGAP overexpressing or silencing treatment. The CD4 expression in **g** NCI-H226 and **h** SK-MES-1 subcutaneous tumor with CD4+ T cells of TAGAP overexpressing or silencing treatment via IF assay (Scale bar = 50 µm). The comparison of apoptosis cell number in **i** NCI-H226 and **j** SK-MES-1 subcutaneous tumor with CD4+ T cells of TAGAP overexpressing or silencing treatment (Scale bar = 50 µm). IF: Immunofluorescence. *P < 0.05, **P < 0.01, ***P < 0.001

Finally, we investigated whether CD4+ T cells of overexpression or silencing TAGAP could affect tumor progression in vivo. For NCI-H226 mouse xenograft tumor model, overexpression of TAGAP on CD4+ T cells inhibited tumor volume and weight; while, TAGAP silencing on CD4+ T cells accelerated tumor progression and increased weight in vivo (Fig. 7 a-c). And the effect of TAGAP in CD4+ T cells on SK-MES-1 in vivo was consistent with NCI-H226 model (Fig. 7 d-f). Moreover, a higher expression of CD4 in TAGAP (ov) group and less expression of CD4 in the TAGAP (si) group in comparison with their corresponding NC group were observed (Fig. 7 g-h). And more TUNEL-positive cell was found in TAGAP (ov) group; while, the TAGAP (si) group had a less TUNEL-positive cell (Fig. 7 i-j). The above results collectively suggested TAGAP could inhibit tumor progression in vivo by increasing the infiltration and toxicity of CD4+ T cells.

### TAGAP regulated CD4+ T cell differentiation by modulating the expression of c-Rel

**Fig. 8:**
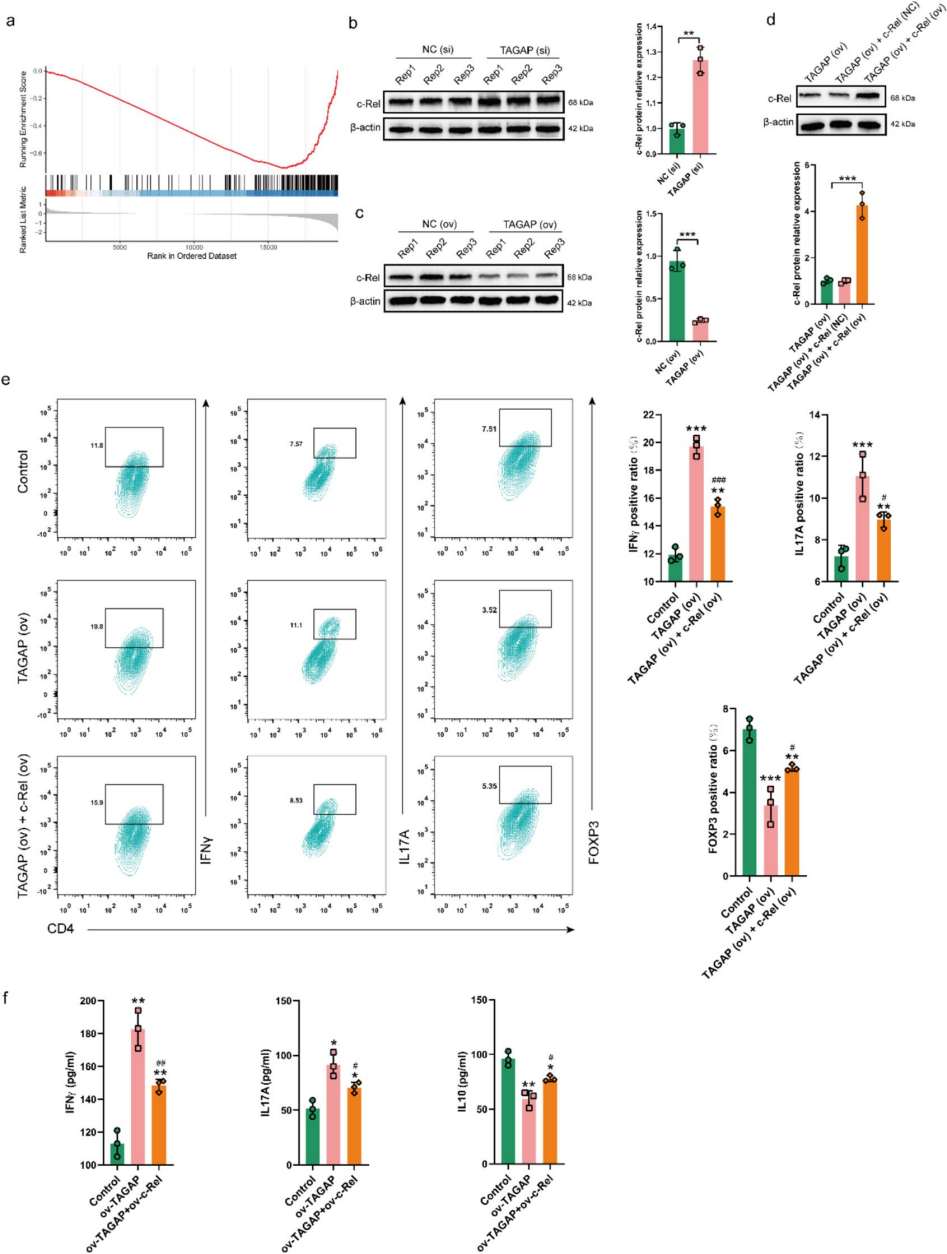
TAGAP participated in the differentiation of CD4+ T cells by regulating the expression of c-Rel. **a** GESA analysis of TAGAP based on the TAGAP profile. c-Rel protein expression level in TAGAP **b** silencing and **c** overexpressing CD4+ T cells. **d** The c-Rel protein expression in TAGAP overexpressing CD4+ T cell after c-Rel (ov) treatment. **e** The percentages of Th1 (CD4^+^ and IFN-γ^+^), Th17 (CD4^+^ and IL17A^+^), and Treg (CD4^+^ and FOXP3^+^) changes after rescuing c-Rel expression in CD4+ T cell overexpressing TAGAP. **P < 0.01, ***P < 0.001 versus Control. ^#^*P* < 0.05, ^###^*P* < 0.001 versus TAGAP (ov). **f** The cytokine levels of IFN-γ, IL17A and IL10 change after rescuing c-Rel expression in CD4+ T cell overexpressing TAGAP. *P < 0.05, **P < 0.01 versus Control. ^#^*P* < 0.05, ^##^*P* < 0.01 versus TAGAP (ov)

Through GSEA analysis of TAGAP, the enriched result revealed that TAGAP mainly negatively regulated the nuclear factor κB (NF-κB) signaling pathway (Fig. 8a). As a previous study, c-Rel, as a subunit of NF-κB, regulates the transcriptional landscape of activated Tregs and restricts its anti-tumor responses of CD4+ T cells [55]. In our result, c-Rel expression was up-regulated and down-regulated in TAGAP (si) and TAGAP (ov) groups, respectively (Fig. 8b-c). After c-Rel expression was rescued by c-Rel (ov) plasmid (Fig. 8d), the rescued c-Rel expression could partly abolish the TAGAP effect on CD4+ T cell differentiation which was represented by inhibiting the proportions of Th1 and Th17 cells, increasing the Treg cell proportion (Fig. 8e). Moreover, rescued c-Rel partially counteracted the promoting effect of TAGAP on IFNγ and IL17A production, while partially promoting IL10 production (Fig. 8f). The above result suggests TAGAP could regulate the differentiation of CD4+ T cells by modulating c-Rel, thereby participating in the regulation of the anti-tumor activity of CD4+ T cells.

## Discussion

Exploring reliable drug targets for treating LUSC is a reliable way to improve the prognosis of LUSC [56, 57]. Unveiling the immunosenescence mechanisms of T cells in cancer is pivotal for screening potential targeted therapeutics [17]. In our study, we compared the mutation characteristics of several common therapeutic agents of NSCLC treatment in LUSC, like *EGFR*, *ALK*, *ROS1*, *BRAF, KARS*, *RET* and *MET*. And the mutation frequency of *ROS1* was the highest among the above genes in LUSC. Next, by comparing the expression profiles of *ROS1*^(wt)^ and *ROS1*^(mut)^, the harvested DEGs were utilized for IRGs screening via WGCNA and prognosis signature building via IRGsP. Based on the signature, the immune milieu in different risk subgroups had been revealed, as well as the related chemotherapy sensibility, immune checkpoint expression, and IPS. Finally, TAGAP was identified as the hub gene among the IRGs and showed abundant expression in CD4+ T cells of LUSC samples. Furthermore, overexpression of TAGAP in CD4+ T cells enhanced its production of cytokines, promoted LUSC cell apoptosis, and inhibited its migration level in vitro. CD4+ T cells with TAGAP overexpressing could also inhibit the progression of tumors due to the rejuvenation of CD4+ T cells’ function in vivo. Our result revealed that TAGAP might be a viable immunosenescence target for the treatment of LUSC.

ROS1 encodes a cellular receptor tyrosine kinase (RTK) containing a transmembrane region, an N-terminal extracellular domain and a C-terminal intracellular tyrosine kinase domain [58]. It was first reported in NSCLC in 2007 [59] and after that, ROS1 became an established therapeutic target of lung cancer [9]. In our present study, *ROS1* mutation was more frequent than other genes in LUSC suggesting ROS1 may involve more potential mechanisms related to LUSC. To explore the potential mechanism related to LUSC, the LUSC samples of TCGA were separated into *ROS1* ^(wt)^ and *ROS1* ^(mut)^ subgroups to conduct the difference analysis. According to the enrichment results, the terms of chemokine signaling pathway or cytokine-cytokine receptor interaction activities were enriched by most DEGs. Moreover, the DEGs have mainly participated in biological processes of signaling transduction and immune response, as well as the molecular function related to protein binding. These results suggested the DEGs related to *ROS1* mutation were not only mainly involved in the immune response related to protein binding such as chemokine or cytokine receptors.

The heterogeneity of TIME is an obstacle to many tumors’ treatment, which is largely caused by the different levels of immune cell infiltration [60]. And different immune infiltration is highly associated with cancer prognosis. A prognosis risk module constructed by DEGs quested by comparing the samples of high- and low-immune infiltration could more accurately evaluate the prognosis of patients with ovarian cancer [61]. Other immune-related prognosis models were also reported in hepatocellular carcinoma [44], colon cancer [36] and NSCLC [62, 63] whose ability to predict the outcome of the corresponding tumor was all better than the clinical TNM staging method. With the same aim to build a more reliable and accurate risk signature of LUSC, the IRGs from DEGs of *ROS1* ^(wt)^ and *ROS1* ^(mut)^ LUSC subgroups were utilized for the risk signature construction which could more accurately evaluate the prognosis of LUSC. More importantly, the time-dependent ROC curve of risk signature was better than the traditional TNM classification and the AUC values for OS evaluation at 1-, 3- and 5-years were all > 0.7. In addition, the nomogram combined with risk signature and traditional clinical information would help to achieve a personalized prediction of LUSC outcomes. These results suggesting the risk signature constructed by IRGs could be realized in the personalized evaluation of LUSC.

Through comparing the levels of immune cell infiltration within the low- and high-risk subgroups, the levels of NK, CD8+ T and follicular helper T cells were decreased and plasma, CD4+ memory resting T cells and macrophage M2 cells were increased in the high-risk subgroup. Among them, NK cells have been reported to demonstrate anti-tumor properties by producing a large number of cytokines like interferon-γ [64]. In addition, CD8+ T cells also known as the CD8+ cytotoxic T lymphocytes. And CD8+ T cells mainly exert the roles of cytotoxicity and anti-tumor [65]. And cells of follicular helper T had been identified as the vital type to activate the immune response [61]. The plasma cells located in tumors or in tumor-draining lymph nodes have a crucial effect on anti-tumor immune responses as well [66]. The memory CD4+ T cell has been reported as an independent index for NSCLC metastasis patients [67]. M2 macrophage cells could contribute to tumor carcinogenesis and less M2 macrophage infiltration always was associated with poorer OS of specific cancers [68]. In the present study, the above anti-tumor immune cells decreased in the high-risk subgroup may be one reason for the poor outcome of LUSC.

With the increasing understanding of tumor immune mechanisms, the treatment of NSCLC has shifted from adjuvant chemotherapy and targeted therapy to immunotherapy. Especially, the discovery of immune checkpoint inhibitors (ICIs) (like anti-PD1 and anti-CTLA4) has brought promising progress in advanced NSCLC patients with better outcomes [69, 70]. Herein, the genes of PDCD1, CTLA4 and PDCD1LG2 expressions were compared between the low- and the high-risk subgroups and found PDCD1, CTLA4 and PDCD1LG2 expressions were all significantly higher in the high-risk subgroup. In addition, the response and efficacy of ICIs’ application could be evaluated via IPS [71]. In our results, we found the IPS of the low-risk subgroup was lower than that within the high-risk subgroup in the application of anti-CTLA4. These results suggested the ICI therapy of CTLA4 might be beneficial for the high-risk subgroup of LUSC. Although the application of ICIs is of crucial significance for NSCLC therapy, only a part of cancer patients can respond to immunotherapy [72]. Next, the sensitivities of common chemotherapy agents were compared between high- and low-risk subgroups of LUSC. It is worth noting that the sensitivities of Gefitinib, Paclitaxel, and Mitomycin.C were increased in the high-risk subgroup and Sunitinib sensitivity was lower in the high-risk subgroup, suggesting Sunitinib might be a potentially effective chemotherapy drug for the high-risk subgroup of LUSC. Finally, considering that application of chemotherapy drugs is easy leading to the emergence of drug resistance and limiting its application, we screened some potential chemotherapy agents according to the hub genes of IRGs. In the results, Nelarabine, Chelerythrine, Imexon, Hydroxyurea, Chlorambucil, Uracil mustard, Isotretinoin, and Fluphenazine may be the effective drug for high-risk LUSC patients.

Exploring new pathogenic targets or molecular mechanisms of LUSC is expected to provide more strategies for LUSC management. Here, via difference and sc-seq analysis of LUSC, TAGAP was found in all cell types containing epithelial cells, fibroblasts, B cells, monocytes, neutrophils, and T cells. However, its expression was more abundant in immune cells, especially T cells and neutrophils. According to previous studies, TAGAP encodes members of the Rho GTPases superfamily, which is involved in the Rho GTPase cycle and responsible for T cell response to stimuli and influencing T cell differentiation [73, 74]. In addition, as the activator of Rho GTPases, TAGAP could exhibit certain effects on autoimmune diseases by overactivation of T cells [75]. Therefore, the lower TAGAP expression, the worse the prognosis of LUSC, and the loss of T cell function may be the key factor. To validate this hypothesis, we conducted a series of experiments based on CD4 T cells in vitro and in vivo. As expected, overexpression of TAGAP on CD4+ T cells could enhance its activity, manifested by increased cytotoxicity, weakened migration ability of co-cultured LUSC cells, increased apoptosis in vitro and significant inhibition of the volume and size of transplanted tumors in vivo. Moreover, more pronounced CD4 expression and increased apoptosis of cells could be observed in xenograft tumors. Moreover, TAGAP could promote the differentiation of CD4+ T cells into Th1/Th17 cells with pro-inflammatory and anti-tumor effects, while inhibiting their differentiation into Tregs with inhibitory immune responses. The above results indicated that TAGAP exerted its anti-tumor effect on CD4+ T cells by affecting their differentiation into Th1/Th17 cells. Immunosenescence drives T cells from quiescence to a terminally differentiated state, characterized by reduced lineage plasticity and a diminished capacity to respond to novel antigenic challenges [16]. Therefore, the observed TAGAP enhanced function of CD4+ T cells may represent a mechanism counteracting immunosenescence.

NF-κB pathway has been demonstrated as a hallmark of immunosenescence in the immune system [27]. Abnormal activation of NF-κB is often associated with the development of tumors, including NSCLC [76, 77]. In this study, we found that TAGAP could negatively regulate the activity of NF-κB via GSEA analysis. There are two pathways for NF-κB activation: canonical and non-canonical pathways. For the canonical pathway, the NF-κB heterodimer composed of p50 and p65 or p50 and c-Rel is mainly involved in immune response and cell survival. And c-Rel activity in CD4+ T cells could restrict their anti-tumor responses and promote its differentiation into Tregs and chemical c-Rel inhibition could reduce melanoma growth [55], suggesting c-Rel might harm CD4+ T immune response. In our study, overexpression of TAGAP in CD4+ T cells could inhibit c-Rel expression while TAGAP silencing up-regulated expression of c-Rel. Moreover, upregulating c-Rel in CD4+ T cells with TAGAP overexpression partially weakened the activation effect of TAGAT on CD4+ T cells toward Th1/Th17 differentiation, facilitating their differentiation into Tregs. Based on the above results, it can be inferred that TAGAP activated CD4+ T cells by regulating c-Rel.

This study presents several limitations that warrant acknowledgment. First, further investigation into the mechanisms driving TAGAP downregulation in CD4+ T cells within the LUSC TIME is crucial. Understanding these upstream processes could inform preventative strategies targeting TAGAP related LUSC development. Second, while this study focused on c-Rel, a crucial regulator of CD4+ T activation and differentiation, a more comprehensive analysis of TAGAP's downstream effects is needed. Employing transcriptional sequencing could elucidate the broader impact of TAGAP on CD4+ T cell function. In addition, the senescence biomarkers of CD4+ T cells after TAGAP targeting should be further investigated in the future. Finally, exploring the role of TAGAP in other immune cell populations within the LUSC context could provide valuable insights for translating TAGAP related findings into clinical applications.

In conclusion, a LUSC prognostic signature constructed with TAGAP as the key gene could reliably distinguish the high-/low-risk LUSC populations. In addition, TAGAP, as a c-Rel suppressor in CD4+ T cells could promote their differentiation into Th1/Th17 cells and inhibit their differentiation into Tregs, thereby rejuvenating the anti-tumor ability of CD4+ T cells.

## Supplementary Information

Below is the link to the electronic supplementary material.

Supplementary file1 (DOCX 1270 KB)

## Data Availability

The datasets used and/or analyzed during the current study are available from the TCGA/GEO database and the corresponding author upon reasonable request.
