# Supplementary material for: Harnessing TAGAP to improve immunotherapy for lung squamous carcinoma treatment by targeting c-Rel in CD4+ T cells
Source: Cancer Immunol Immunother. 2025 Feb 25;74(4):114. doi: 10.1007/s00262-025-03960-1 (PMC11861500; doi:10.1007/s00262-025-03960-1)
Supplement: Supplementary file 1 — Supplementary file1 (DOCX 1270 KB) [file 262_2025_3960_MOESM1_ESM.docx]

**Figure S1**


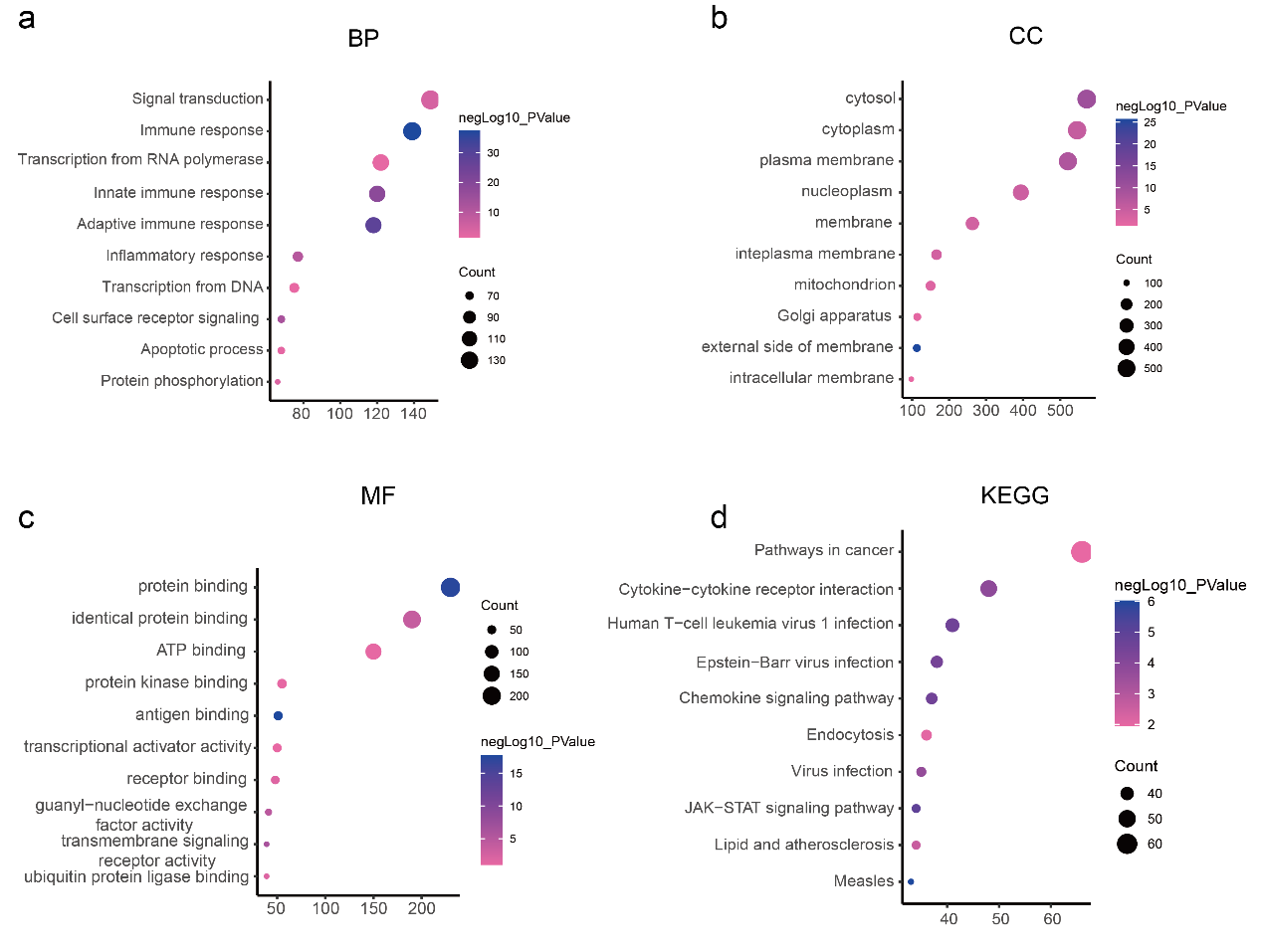


**Figure S1. The enrichment analysis of DEGs.** GO enrichment analysis of DEGs between *ROS1*^MUT^ and *ROS1*^WT^ LUSC samples in terms of (**a**) BP, (**b**) CC and **(c)** MF. **(d).** KEGG enrichment analyses of DEGs between *ROS1*^MUT^ and *ROS1*^WT^ LUSC samples. LUSC: lung squamous cell carcinoma. GO: gene ontology. DEGs: differentially expressed genes. BP: biological process. CC: cellular component. MF: molecular function. KEGG: Kyoto Encyclopedia of Genes and Genomes.

**Figure S2**


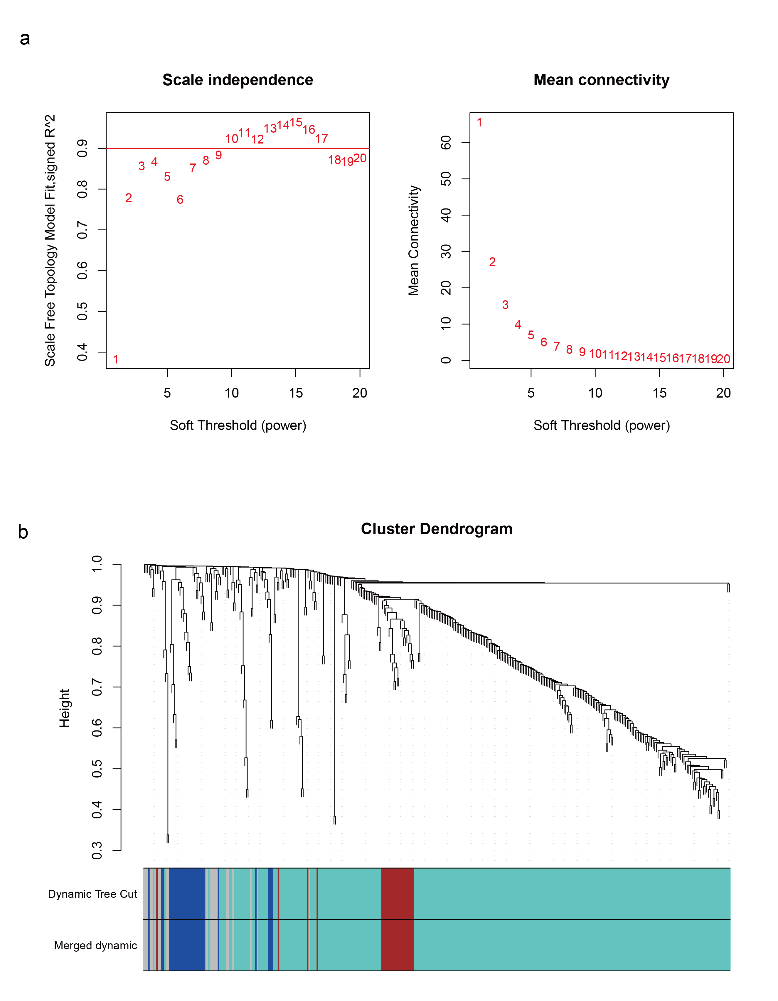


**Figure S2. IRGs screening via WGCNA. (a).** Analysis of the scale‐free fit index and mean connectivity for various soft‐thresholding powers. **(b).** The hierarchical cluster analysis identified the coexpression clusters based on the topological overlap. Each module corresponds to one color and the gray module contains unassigned genes. IRGs: immune-related DEGs. WGCNA: weighted correlation network analysis.

**Figure S3**


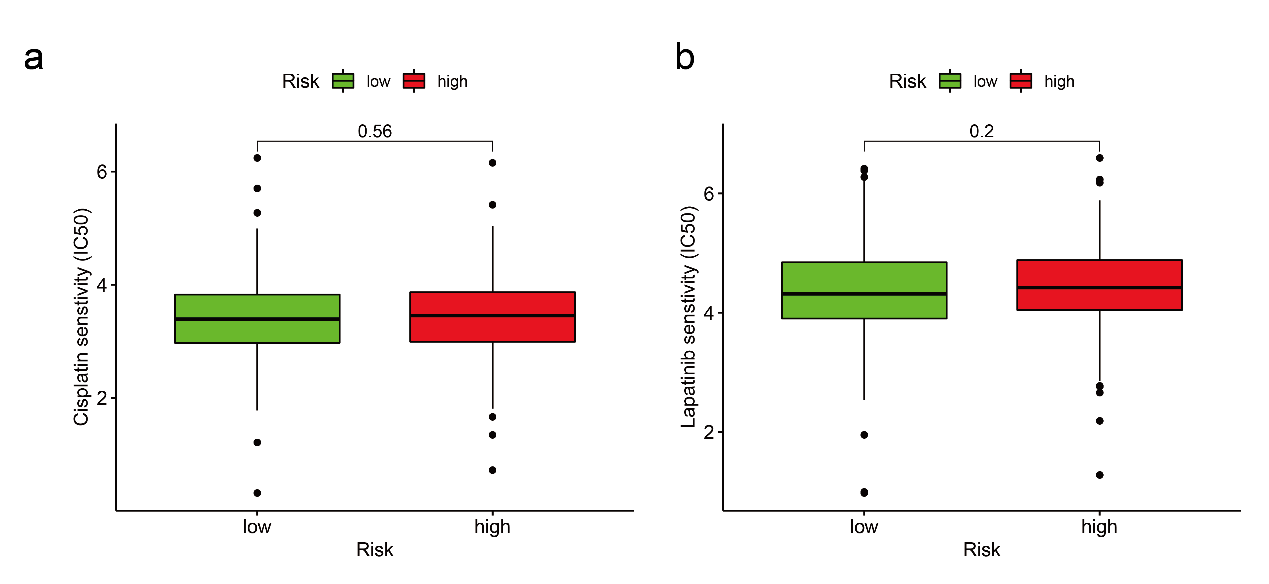


**Figure S3. Comparison of chemotherapy sensitivities between high-/low-risk subgroups.** The comparison of **(a)** Cisplatin and **(b)** Lapatinib sensitivities between the different risk subgroups.

**Figure S4**


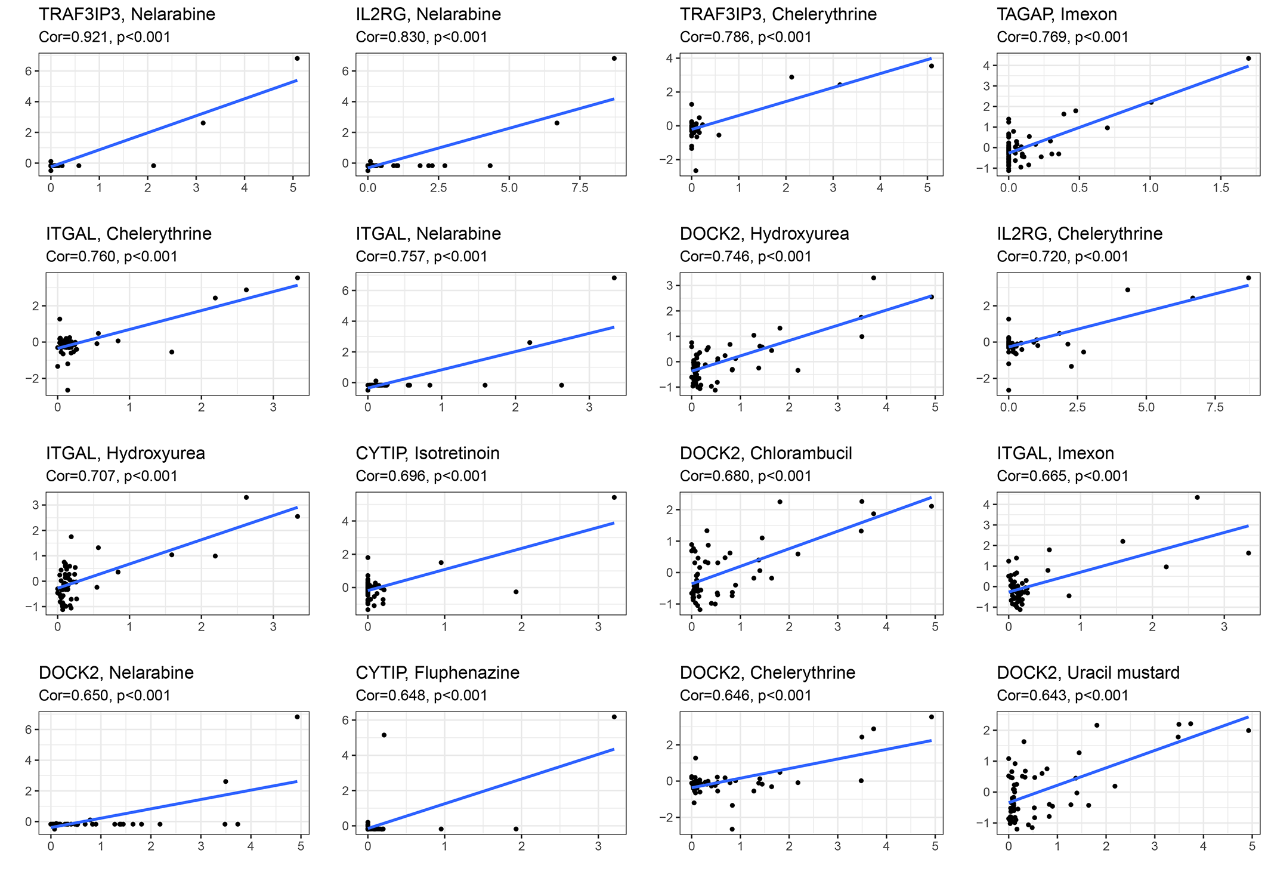


**Figure S4. The correlation analysis of chemo-drugs IC50 with IRGs (nodes ≥ 5) expressions. IC50: half maximal inhibitory concentration.**

**Figure S5**


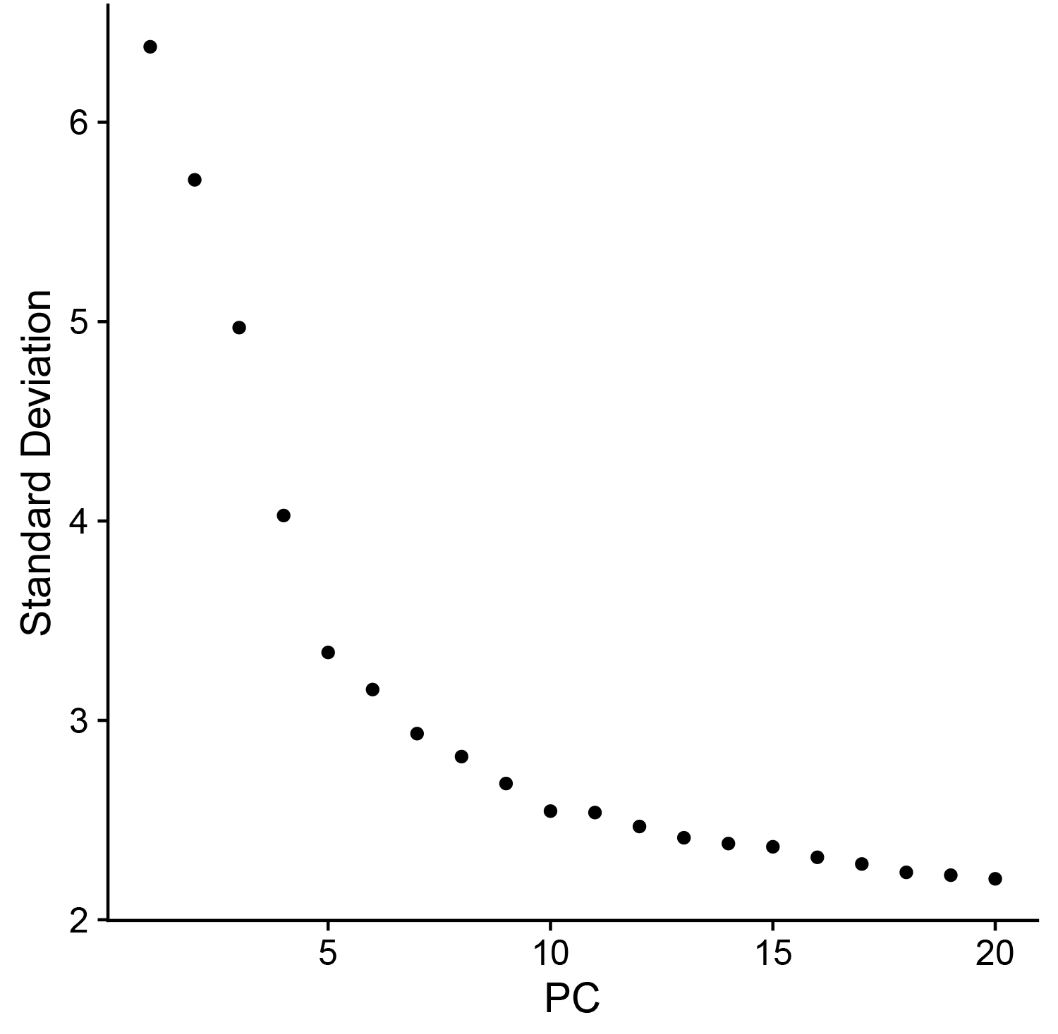


**Figure S5. The Scree Plot analysis to screen the rational number of principal components.**

**Figure S6**
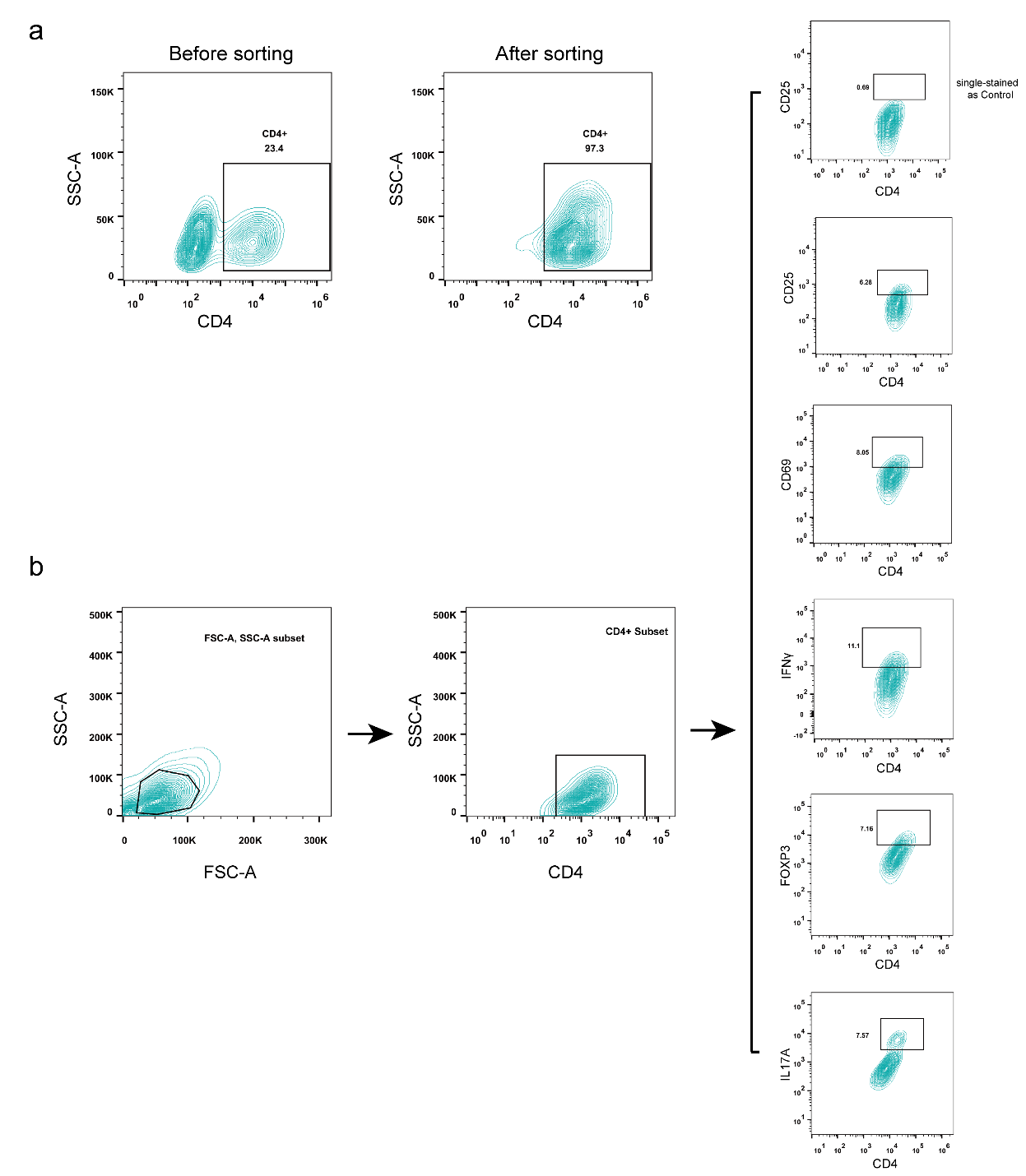


**Figure S6. Detection of CD4 + T cells by flow cytometry. (a).** The purity of CD4+ T cells before and after sorting. **(b).** Schematic diagram of Control group set and gating strategy for CD4+ T cell subpopulation.

Table S1. Immune infiltration scores for LUSC samples.

| ID | | StromalScore | ImmuneScore | ESTIMATEScore |
| --- | --- | --- | --- | --- |
| TCGA-34-5234 | 1439.84452 | | 2402.39828 | 3842.242806 |
| TCGA-96-7544 | 964.3416 | | 1300.31372 | 2264.655316 |
| TCGA-85-A513 | 849.141144 | | 2552.93974 | 3402.080882 |
| TCGA-85-8584 | -126.09066 | | 1863.74125 | 1737.650594 |
| TCGA-85-A511 | 662.136934 | | 1273.68987 | 1935.8268 |
| TCGA-66-2789 | 570.416864 | | 937.974929 | 1508.391793 |
| TCGA-22-0940 | 422.245333 | | 2133.37734 | 2555.622678 |
| TCGA-96-A4JL | 417.629567 | | 2491.33385 | 2908.963413 |
| TCGA-77-8136 | 775.154666 | | 2050.57997 | 2825.734633 |
| TCGA-63-5131 | -686.88196 | | 480.716455 | -206.165505 |
| TCGA-90-7964 | 44.0378281 | | 988.217197 | 1032.255025 |
| TCGA-85-A5B5 | -695.27478 | | 27.1535057 | -668.121275 |
| TCGA-37-3789 | -166.63575 | | 1525.86191 | 1359.22616 |
| TCGA-O2-A5IB | -1451.8071 | | -827.05831 | -2278.86542 |
| TCGA-58-8390 | 271.813271 | | 695.142637 | 966.9559077 |
| TCGA-66-2777 | 156.566634 | | 1552.18842 | 1708.755056 |
| TCGA-77-8140 | -1176.259 | | 80.3481484 | -1095.91082 |
| TCGA-98-A538 | -247.01296 | | 739.052124 | 492.0391606 |
| TCGA-66-2785 | 149.963286 | | 2235.40742 | 2385.370702 |
| TCGA-43-5670 | -1119.8112 | | -446.23908 | -1566.05028 |
| TCGA-22-5472 | -186.35779 | | 1104.16778 | 917.8099938 |
| TCGA-60-2704 | 1528.50965 | | 2172.30816 | 3700.817807 |
| TCGA-77-6843 | -1304.1614 | | -477.21854 | -1781.37994 |
| TCGA-63-A5MG | -41.776357 | | 1288.61277 | 1246.836414 |
| TCGA-66-2734 | 479.16407 | | 1557.90681 | 2037.070878 |
| TCGA-43-2581 | 824.644486 | | 2192.91951 | 3017.563999 |
| TCGA-52-7811 | 101.589185 | | 1009.86 | 1111.449185 |
| TCGA-85-8287 | 1091.28001 | | 2006.60258 | 3097.882585 |
| TCGA-85-8355 | 426.283431 | | 2768.29373 | 3194.577159 |
| TCGA-94-7033 | -1500.0343 | | 290.835841 | -1209.19842 |
| TCGA-60-2697 | 1011.79754 | | 2778.93426 | 3790.7318 |
| TCGA-77-8145 | 571.265518 | | 384.046131 | 955.3116495 |
| TCGA-85-7844 | 515.406003 | | 899.717426 | 1415.123429 |
| TCGA-39-5040 | -740.29485 | | 221.784507 | -518.51034 |
| TCGA-66-2795 | 714.354801 | | 1402.959 | 2117.313803 |
| TCGA-33-4586 | -549.81601 | | 117.42187 | -432.394141 |
| TCGA-NC-A5HH | -1126.5005 | | 46.5832386 | -1079.91731 |
| TCGA-22-1002 | 599.363453 | | 1396.98702 | 1996.35047 |
| TCGA-60-2695 | -277.98608 | | 937.804994 | 659.8189165 |
| TCGA-77-A5G8 | -328.53774 | | 772.132151 | 443.5944155 |
| TCGA-63-A5MP | -196.5506 | | 1693.46699 | 1496.916386 |
| TCGA-22-0944 | -962.48841 | | -8.3654737 | -970.853879 |
| TCGA-21-1081 | -43.913491 | | 0.77426284 | -43.1392279 |
| TCGA-O2-A52V | -189.93259 | | 1023.61235 | 833.6797598 |
| TCGA-33-AASB | -1924.3313 | | -457.06363 | -2381.3949 |
| TCGA-63-5128 | -549.38317 | | -144.72852 | -694.111687 |
| TCGA-96-8170 | -112.00531 | | 1105.43236 | 993.4270474 |
| TCGA-63-A5MW | 263.498802 | | -27.437083 | 236.0617183 |
| TCGA-37-3792 | 683.407972 | | 2757.36592 | 3440.773893 |
| TCGA-56-A4BX | -1910.4301 | | -897.27446 | -2807.70456 |
| TCGA-56-8083 | -863.35427 | | -869.57432 | -1732.92859 |
| TCGA-98-A53H | 1153.72517 | | 2307.61786 | 3461.34303 |
| TCGA-94-A4VJ | -283.54361 | | 726.154092 | 442.6104771 |
| TCGA-43-7658 | -1252.7695 | | 1179.53427 | -73.2352412 |
| TCGA-NC-A5HN | -1345.991 | | -89.249844 | -1435.24085 |
| TCGA-63-7021 | 215.343496 | | 1455.47448 | 1670.817971 |
| TCGA-22-5471 | -124.69053 | | 1677.65054 | 1552.960015 |
| TCGA-77-8133 | -333.86107 | | 1753.39246 | 1419.531396 |
| TCGA-37-A5EM | 799.201182 | | 1484.20868 | 2283.40986 |
| TCGA-85-8071 | -1068.9893 | | 173.491781 | -895.497544 |
| TCGA-NC-A5HP | 145.880736 | | -0.0739028 | 145.8068331 |
| TCGA-85-A4PA | 179.106317 | | 3437.633 | 3616.739317 |
| TCGA-33-4582 | -1108.8481 | | -73.752171 | -1182.60024 |
| TCGA-43-2578 | -424.94647 | | 796.375911 | 371.4294448 |
| TCGA-34-A5IX | -920.6657 | | 694.979105 | -225.686599 |
| TCGA-56-8504 | -23.716749 | | 1023.4053 | 999.6885525 |
| TCGA-18-5595 | -1088.055 | | -394.6442 | -1482.69922 |
| TCGA-21-5784 | 150.618308 | | 1689.55013 | 1840.168438 |
| TCGA-90-A4ED | -72.340367 | | 604.708264 | 532.3678971 |
| TCGA-MF-A522 | -1356.9922 | | -97.225156 | -1454.21731 |
| TCGA-46-3768 | -588.83671 | | 464.417665 | -124.419042 |
| TCGA-77-7335 | 1847.15845 | | 2501.41882 | 4348.577276 |
| TCGA-56-A4BY | -688.57556 | | 422.221914 | -266.353645 |
| TCGA-85-8070 | -1820.8638 | | -679.40288 | -2500.26667 |
| TCGA-56-7579 | -317.06159 | | 484.627494 | 167.5659081 |
| TCGA-NC-A5HG | -1464.1465 | | -197.67843 | -1661.82491 |
| TCGA-56-8305 | -294.15424 | | 244.371115 | -49.7831301 |
| TCGA-60-2703 | 278.854201 | | 228.961527 | 507.8157274 |
| TCGA-39-5036 | -1566.0589 | | 458.444513 | -1107.61439 |
| TCGA-33-6737 | 82.7759388 | | 1099.539 | 1182.314935 |
| TCGA-34-2608 | 1249.03584 | | 1964.97481 | 3214.010655 |
| TCGA-39-5024 | -894.37484 | | 865.027706 | -29.3471352 |
| TCGA-77-7142 | -183.49627 | | 623.923491 | 440.4272156 |
| TCGA-85-8350 | 142.329885 | | 999.923376 | 1142.253261 |
| TCGA-O2-A52Q | 278.536723 | | 2262.10217 | 2540.638891 |
| TCGA-68-8250 | -207.64852 | | 1219.49313 | 1011.844611 |
| TCGA-70-6723 | -1473.9594 | | -405.28885 | -1879.2482 |
| TCGA-68-8251 | 584.805021 | | 1290.17444 | 1874.979462 |
| TCGA-85-7697 | -161.29916 | | 959.026779 | 797.7276212 |
| TCGA-66-2756 | -507.67911 | | 351.388418 | -156.290692 |
| TCGA-85-6561 | 1389.83511 | | 1979.89636 | 3369.731465 |
| TCGA-94-7943 | -667.86989 | | 636.286696 | -31.5831948 |
| TCGA-22-1011 | 224.937361 | | 1940.86952 | 2165.806877 |
| TCGA-85-8664 | -1730.0859 | | -206.93167 | -1937.0176 |
| TCGA-63-A5MH | -711.43264 | | -68.286678 | -779.719321 |
| TCGA-94-8490 | -1296.4396 | | -408.10244 | -1704.54207 |
| TCGA-85-8481 | 501.940065 | | 1151.71898 | 1653.659041 |
| TCGA-60-2716 | -1433.9114 | | -549.18337 | -1983.09477 |
| TCGA-O2-A52S | -909.25588 | | 979.221633 | 69.96574918 |
| TCGA-51-4080 | -1086.2262 | | -20.814991 | -1107.04123 |
| TCGA-22-5485 | -557.82692 | | 442.246846 | -115.580072 |
| TCGA-85-6175 | 1689.47779 | | 1939.971 | 3629.4488 |
| TCGA-85-8276 | 921.887405 | | 1788.47532 | 2710.362729 |
| TCGA-77-8008 | 1562.18853 | | 1683.48985 | 3245.678378 |
| TCGA-39-5028 | 452.8687 | | 1356.9752 | 1809.843902 |
| TCGA-34-5928 | 1121.77099 | | 1016.79334 | 2138.564332 |
| TCGA-43-6773 | 1232.18055 | | 2360.8709 | 3593.051453 |
| TCGA-63-A5MI | -1010.4066 | | -274.05282 | -1284.45945 |
| TCGA-63-A5ML | -315.96485 | | 581.980193 | 266.0153466 |
| TCGA-39-5030 | 1083.89901 | | 2685.67756 | 3769.576575 |
| TCGA-51-4079 | -62.315975 | | 951.411955 | 889.0959808 |
| TCGA-22-5491 | -1272.9625 | | 389.117056 | -883.845448 |
| TCGA-66-2744 | 676.293026 | | 2572.43581 | 3248.728837 |
| TCGA-33-4547 | 34.5770188 | | 886.73516 | 921.3121784 |
| TCGA-52-7810 | -660.80983 | | -87.751803 | -748.561636 |
| TCGA-77-7139 | -559.10116 | | 361.092471 | -198.008687 |
| TCGA-66-2800 | 192.249191 | | 1452.21387 | 1644.463061 |
| TCGA-33-4566 | -87.04564 | | 1734.26358 | 1647.21794 |
| TCGA-90-7766 | -798.31497 | | 1520.46033 | 722.1453516 |
| TCGA-60-2707 | 547.750865 | | 1116.59962 | 1664.350489 |
| TCGA-56-8628 | 666.17551 | | 1891.03964 | 2557.215149 |
| TCGA-85-8666 | 515.325803 | | 1221.56182 | 1736.887626 |
| TCGA-60-2721 | 348.689237 | | 1813.90967 | 2162.598905 |
| TCGA-22-4596 | 1296.02629 | | 2740.9813 | 4037.007587 |
| TCGA-33-A5GW | -1555.3968 | | -408.27724 | -1963.67402 |
| TCGA-60-2715 | 1419.6888 | | 2249.26454 | 3668.953337 |
| TCGA-85-8351 | -461.23524 | | 584.169619 | 122.9343828 |
| TCGA-58-A46M | -638.83572 | | 1167.34769 | 528.511972 |
| TCGA-37-4129 | -1082.5677 | | -794.07054 | -1876.63822 |
| TCGA-21-1075 | -1198.9072 | | -578.48007 | -1777.38725 |
| TCGA-43-7656 | 196.532198 | | 1853.66385 | 2050.196048 |
| TCGA-58-8388 | 397.240757 | | 1418.47479 | 1815.715547 |
| TCGA-98-A53J | -1082.5749 | | 74.3875228 | -1008.18739 |
| TCGA-85-7696 | -291.70407 | | 567.117611 | 275.4135376 |
| TCGA-34-5231 | -548.60793 | | 767.782776 | 219.1748461 |
| TCGA-34-5929 | -384.7193 | | 746.362579 | 361.6432767 |
| TCGA-22-5492 | 196.727144 | | 1957.76106 | 2154.488203 |
| TCGA-98-A53C | 1415.47281 | | 2896.41511 | 4311.887926 |
| TCGA-94-8491 | -609.20822 | | 1373.44576 | 764.2375427 |
| TCGA-90-7767 | 680.755293 | | 1594.4206 | 2275.175891 |
| TCGA-96-8169 | 56.1116119 | | 1137.2661 | 1193.377709 |
| TCGA-34-2596 | -193.27194 | | 1000.54218 | 807.2702405 |
| TCGA-56-A62T | -204.30354 | | 637.654788 | 433.3512459 |
| TCGA-21-1071 | 87.4257288 | | 923.012371 | 1010.4381 |
| TCGA-22-5480 | 19.5715577 | | 1840.57384 | 1860.145395 |
| TCGA-77-A5GH | 562.441699 | | 2251.3456 | 2813.787301 |
| TCGA-L3-A4E7 | -347.3558 | | 1101.43889 | 754.0830912 |
| TCGA-60-2723 | 267.557939 | | 1606.41815 | 1873.976086 |
| TCGA-37-3783 | 435.157662 | | 2077.10699 | 2512.264655 |
| TCGA-66-2763 | -397.08043 | | 864.008697 | 466.9282631 |
| TCGA-63-6202 | 772.83129 | | 2445.16245 | 3217.993738 |
| TCGA-L3-A524 | -547.93112 | | -10.40713 | -558.338252 |
| TCGA-98-8021 | -32.420405 | | 465.698792 | 433.2783869 |
| TCGA-77-A5GF | -345.65154 | | 626.552617 | 280.9010807 |
| TCGA-66-2787 | 184.883977 | | 1510.49252 | 1695.376497 |
| TCGA-77-8138 | 324.039718 | | 1247.0146 | 1571.054314 |
| TCGA-63-A5MR | -880.11829 | | 501.79343 | -378.324856 |
| TCGA-66-2768 | -443.40452 | | 1433.37855 | 989.9740277 |
| TCGA-77-8146 | -276.74331 | | 729.626593 | 452.8832788 |
| TCGA-43-A56V | -647.67858 | | 417.617965 | -230.060618 |
| TCGA-22-1005 | 855.426434 | | 2870.50682 | 3725.933254 |
| TCGA-66-2780 | 368.217496 | | 1074.51637 | 1442.733866 |
| TCGA-77-8156 | 550.571604 | | 2084.20962 | 2634.781222 |
| TCGA-66-2790 | 197.99229 | | 1179.60349 | 1377.595784 |
| TCGA-98-8022 | 40.0915869 | | 1428.88243 | 1468.97402 |
| TCGA-58-A46L | -63.554601 | | 1061.13489 | 997.5802872 |
| TCGA-O2-A52N | -533.17546 | | 936.59172 | 403.4162628 |
| TCGA-63-7022 | 1622.57382 | | 2355.17159 | 3977.745405 |
| TCGA-37-4135 | -424.20196 | | 1017.64123 | 593.4392676 |
| TCGA-66-2727 | -247.42371 | | 976.672254 | 729.2485411 |
| TCGA-34-7107 | 568.636681 | | 638.084013 | 1206.720694 |
| TCGA-NC-A5HD | -1234.2657 | | -534.90363 | -1769.16935 |
| TCGA-56-7582 | -269.49069 | | 703.145035 | 433.6543425 |
| TCGA-18-3415 | 298.356864 | | 1428.27725 | 1726.634116 |
| TCGA-56-7580 | -550.44213 | | 494.776463 | -55.6656656 |
| TCGA-33-AASL | -1309.1549 | | 736.06712 | -573.087813 |
| TCGA-43-2576 | 947.765478 | | 1950.00912 | 2897.774596 |
| TCGA-77-A5FZ | 1558.6353 | | 3379.00658 | 4937.641877 |
| TCGA-33-4583 | -1117.0904 | | 733.563079 | -383.527275 |
| TCGA-46-6026 | -1425.7001 | | -189.17155 | -1614.87168 |
| TCGA-18-3414 | -182.44749 | | 1027.41849 | 844.9709991 |
| TCGA-63-A5MY | -1944.6043 | | -736.10087 | -2680.70516 |
| TCGA-56-8082 | -974.62834 | | -121.91461 | -1096.54295 |
| TCGA-60-2712 | 921.29134 | | 2501.7226 | 3423.013937 |
| TCGA-60-2698 | 214.774198 | | 1929.76744 | 2144.541641 |
| TCGA-52-7809 | -761.34193 | | 205.760208 | -555.581727 |
| TCGA-34-5232 | -454.94479 | | 2127.31964 | 1672.374857 |
| TCGA-18-4083 | 46.3359899 | | 674.658223 | 720.9942132 |
| TCGA-37-A5EN | -869.39275 | | 542.837464 | -326.55529 |
| TCGA-85-A510 | -371.91087 | | 1275.2408 | 903.3299275 |
| TCGA-60-2720 | 68.0056974 | | 1678.31173 | 1746.317428 |
| TCGA-60-2710 | 219.598767 | | 1320.11482 | 1539.713587 |
| TCGA-94-8035 | -1083.7108 | | 176.694723 | -907.016073 |
| TCGA-22-4593 | 482.89614 | | 200.165299 | 683.0614398 |
| TCGA-18-3419 | -790.35853 | | 1348.35923 | 558.000703 |
| TCGA-66-2793 | -1636.225 | | -655.88476 | -2292.10979 |
| TCGA-21-1079 | 396.083118 | | 565.767565 | 961.8506837 |
| TCGA-92-8064 | 732.542969 | | 2275.43555 | 3007.978522 |
| TCGA-63-7023 | -713.32332 | | 463.401635 | -249.921688 |
| TCGA-60-2706 | -25.882188 | | 1671.30353 | 1645.421347 |
| TCGA-98-7454 | 1645.36334 | | 2516.35277 | 4161.716104 |
| TCGA-37-4141 | -162.69622 | | 1105.6467 | 942.9504722 |
| TCGA-22-5477 | -827.11247 | | 403.152347 | -423.960128 |
| TCGA-33-4533 | 12.9535306 | | 37.9193504 | 50.87288096 |
| TCGA-77-8128 | -89.491109 | | -576.36461 | -665.855718 |
| TCGA-56-8309 | 749.191039 | | 2792.7055 | 3541.896538 |
| TCGA-56-A5DR | -171.0169 | | 606.678763 | 435.6618667 |
| TCGA-37-4130 | 2.90980656 | | 2712.70453 | 2715.614334 |
| TCGA-58-A46J | -335.70223 | | 1110.17514 | 774.4729097 |
| TCGA-43-6143 | -208.73455 | | 62.3516245 | -146.382925 |
| TCGA-77-A5G6 | -1116.8638 | | -3.2347007 | -1120.09853 |
| TCGA-39-5031 | 194.877352 | | 1267.33301 | 1462.210363 |
| TCGA-90-A59Q | 418.598407 | | 2467.87162 | 2886.47003 |
| TCGA-68-7755 | -138.98368 | | -64.82423 | -203.80791 |
| TCGA-66-2767 | 637.94616 | | 2570.74339 | 3208.689553 |
| TCGA-66-2771 | 290.18482 | | 2351.49843 | 2641.683252 |
| TCGA-85-6560 | 662.816154 | | 1392.69548 | 2055.511636 |
| TCGA-56-8304 | -1088.0004 | | 833.973551 | -254.026826 |
| TCGA-56-7221 | -1282.2291 | | -174.26877 | -1456.49782 |
| TCGA-18-3421 | -318.61086 | | 1336.98168 | 1018.370824 |
| TCGA-51-6867 | -268.44248 | | 705.27441 | 436.8319344 |
| TCGA-NC-A5HL | -657.73483 | | 1551.03644 | 893.3016074 |
| TCGA-77-A5G1 | 2.84744338 | | 1364.71435 | 1367.561797 |
| TCGA-96-7545 | 498.050095 | | 1639.28192 | 2137.332017 |
| TCGA-NC-A5HM | -1180.8631 | | 664.669447 | -516.193685 |
| TCGA-33-4589 | -176.76124 | | 1717.96788 | 1541.20664 |
| TCGA-85-A4QR | -2197.284 | | -743.92684 | -2941.21082 |
| TCGA-60-2711 | -947.17239 | | 653.509523 | -293.662862 |
| TCGA-33-4532 | -1395.9378 | | -187.04822 | -1582.98604 |
| TCGA-NC-A5HJ | 214.070903 | | 2397.84879 | 2611.919689 |
| TCGA-21-5787 | 211.930098 | | 1978.51655 | 2190.446653 |
| TCGA-85-8479 | -1063.9334 | | -240.76635 | -1304.6998 |
| TCGA-34-5240 | 191.855006 | | 658.060049 | 849.9150543 |
| TCGA-77-8148 | -57.958661 | | 456.089319 | 398.1306586 |
| TCGA-56-8626 | -199.94481 | | 669.787495 | 469.842684 |
| TCGA-33-A4WN | -930.85563 | | 374.874901 | -555.980732 |
| TCGA-39-5027 | -1791.4117 | | -401.16477 | -2192.57651 |
| TCGA-56-8503 | 1354.31724 | | 1914.6452 | 3268.962434 |
| TCGA-56-8623 | 1191.96204 | | 2902.98637 | 4094.948412 |
| TCGA-77-6845 | -974.09616 | | -542.47327 | -1516.56943 |
| TCGA-85-A50Z | -784.45756 | | 1190.26065 | 405.8030926 |
| TCGA-77-8144 | -937.48554 | | 436.9511 | -500.53444 |
| TCGA-85-A53L | -678.90827 | | 312.051679 | -366.856595 |
| TCGA-XC-AA0X | -13.541415 | | 1626.64838 | 1613.106962 |
| TCGA-66-2782 | 424.384585 | | 1762.07717 | 2186.46175 |
| TCGA-68-A59I | 425.812177 | | 1424.56049 | 1850.372671 |
| TCGA-77-A5G7 | -1392.4692 | | -53.330099 | -1445.79934 |
| TCGA-90-A4EE | 729.431773 | | 2757.54604 | 3486.977808 |
| TCGA-98-8023 | 727.843681 | | 1874.75528 | 2602.598961 |
| TCGA-34-8456 | -437.2368 | | 543.004249 | 105.7674466 |
| TCGA-56-5897 | 1377.70204 | | 1935.09339 | 3312.795438 |
| TCGA-66-2786 | -17.406883 | | 1462.31595 | 1444.909063 |
| TCGA-39-5037 | -2015.5445 | | -539.09463 | -2554.6391 |
| TCGA-56-6545 | 1366.30218 | | 1882.31933 | 3248.621505 |
| TCGA-NK-A5CT | -1223.056 | | 605.016563 | -618.03943 |
| TCGA-22-1016 | 823.033176 | | 1680.773 | 2503.806179 |
| TCGA-39-5016 | -732.83038 | | 1574.63416 | 841.8037773 |
| TCGA-63-A5MM | 792.380424 | | 2075.11772 | 2867.498142 |
| TCGA-33-4538 | -816.38694 | | 426.477295 | -389.909648 |
| TCGA-63-A5MT | 266.060742 | | 1326.20327 | 1592.264013 |
| TCGA-18-3416 | -378.67837 | | 2186.9413 | 1808.262926 |
| TCGA-66-2753 | 58.9638059 | | 898.344457 | 957.3082624 |
| TCGA-63-7020 | -292.76471 | | 1354.1785 | 1061.413797 |
| TCGA-56-8308 | -48.358486 | | 810.338012 | 761.9795257 |
| TCGA-18-3408 | 103.396536 | | 1152.43004 | 1255.826572 |
| TCGA-NK-A5D1 | -1201.7773 | | -195.97314 | -1397.75047 |
| TCGA-85-6798 | -77.516561 | | -132.86508 | -210.381641 |
| TCGA-22-4599 | 203.98003 | | 2152.60941 | 2356.58944 |
| TCGA-56-A4ZK | -236.09014 | | 1490.95911 | 1254.868969 |
| TCGA-56-7731 | 680.860287 | | 1651.87529 | 2332.735573 |
| TCGA-92-7341 | 208.272057 | | 478.81756 | 687.089617 |
| TCGA-85-8353 | 813.798617 | | 1457.70457 | 2271.503191 |
| TCGA-85-A4QQ | -1197.0009 | | 235.210803 | -961.790104 |
| TCGA-77-6842 | 342.016329 | | 2266.7708 | 2608.787131 |
| TCGA-22-4595 | 460.859403 | | 1370.9528 | 1831.812202 |
| TCGA-33-4587 | -883.03022 | | -136.35124 | -1019.38145 |
| TCGA-56-A4BW | -88.121144 | | 865.138654 | 777.0175104 |
| TCGA-21-1076 | 1134.06156 | | 2553.94443 | 3688.005996 |
| TCGA-6A-AB49 | -1984.2635 | | -190.1288 | -2174.39232 |
| TCGA-63-A5M9 | -311.78922 | | 737.99199 | 426.202774 |
| TCGA-39-5022 | 963.738011 | | 2582.64889 | 3546.386904 |
| TCGA-43-6771 | 234.117585 | | 1023.5573 | 1257.67488 |
| TCGA-98-8020 | 393.645263 | | 1241.44343 | 1635.088697 |
| TCGA-77-7141 | -769.27647 | | 134.842773 | -634.433696 |
| TCGA-92-7340 | 1238.70764 | | 1442.29089 | 2680.998526 |
| TCGA-56-8622 | 230.587013 | | 739.506077 | 970.0930894 |
| TCGA-70-6722 | 693.151473 | | 1410.99319 | 2104.144662 |
| TCGA-43-A56U | 404.358403 | | 2688.2053 | 3092.563702 |
| TCGA-66-2770 | 400.2216 | | 1352.07354 | 1752.295136 |
| TCGA-85-7710 | -649.15157 | | 887.930348 | 238.7787774 |
| TCGA-58-8387 | -453.55257 | | 1855.31321 | 1401.760642 |
| TCGA-85-8352 | -1801.1312 | | -514.23116 | -2315.36239 |
| TCGA-21-5786 | -188.81081 | | 492.197976 | 303.3871691 |
| TCGA-33-AASI | -985.94042 | | 1146.02735 | 160.0869353 |
| TCGA-56-8625 | 1271.52992 | | 2681.81287 | 3953.342796 |
| TCGA-34-2600 | -1330.0069 | | 56.9802872 | -1273.02658 |
| TCGA-60-2726 | -770.23939 | | 1110.61254 | 340.3731498 |
| TCGA-77-8153 | -1190.1616 | | -528.71211 | -1718.87372 |
| TCGA-66-2791 | 610.128746 | | 2047.40383 | 2657.532575 |
| TCGA-68-7756 | -83.460929 | | 609.769238 | 526.3083089 |
| TCGA-94-7557 | -199.3182 | | 694.088052 | 494.7698525 |
| TCGA-56-A5DS | -380.83049 | | 1291.86224 | 911.0317478 |
| TCGA-22-4613 | -214.57211 | | 1595.93047 | 1381.358365 |
| TCGA-77-8130 | 403.114473 | | 326.294159 | 729.408632 |
| TCGA-21-1080 | -694.19774 | | 773.193036 | 78.99529535 |
| TCGA-98-A53I | -97.531984 | | 1673.74888 | 1576.216897 |
| TCGA-34-5236 | 894.426234 | | 1088.46942 | 1982.895654 |
| TCGA-21-5782 | 111.576229 | | 826.48794 | 938.0641695 |
| TCGA-60-2708 | 792.677738 | | 407.121489 | 1199.799227 |
| TCGA-96-A4JK | -360.9889 | | 929.191094 | 568.2021953 |
| TCGA-66-2788 | 917.685188 | | 1690.35548 | 2608.040668 |
| TCGA-56-8624 | 121.226846 | | 727.117968 | 848.3448146 |
| TCGA-22-5473 | -286.4751 | | 553.923624 | 267.4485207 |
| TCGA-21-A5DI | -763.20141 | | 75.6565271 | -687.544884 |
| TCGA-85-8049 | 1090.65659 | | 2242.646 | 3333.302598 |
| TCGA-21-5783 | -95.285097 | | -85.939582 | -181.224679 |
| TCGA-46-3765 | -31.262703 | | 1119.69568 | 1088.432974 |
| TCGA-39-5021 | 48.5260743 | | 1340.52845 | 1389.054529 |
| TCGA-90-6837 | 909.348124 | | 1745.91049 | 2655.258617 |
| TCGA-39-5011 | 45.6302999 | | 2419.85617 | 2465.486473 |
| TCGA-39-5019 | 138.395379 | | 1131.63251 | 1270.027888 |
| TCGA-98-A539 | -264.32801 | | 755.754161 | 491.4261526 |
| TCGA-66-2781 | -88.401498 | | 860.326913 | 771.9254152 |
| TCGA-37-5819 | -442.00444 | | 651.703267 | 209.6988235 |
| TCGA-56-8201 | 1116.79839 | | 2592.08676 | 3708.885147 |
| TCGA-63-A5MB | -783.05193 | | 506.306998 | -276.744937 |
| TCGA-60-2722 | -891.20361 | | -19.807047 | -911.01066 |
| TCGA-85-8048 | 855.647308 | | 2372.46309 | 3228.110396 |
| TCGA-56-7223 | -1296.4405 | | -1167.1328 | -2463.57335 |
| TCGA-43-8115 | 1375.83235 | | 2116.22722 | 3492.059572 |
| TCGA-18-5592 | -680.16383 | | 310.260514 | -369.90332 |
| TCGA-77-7337 | 743.38294 | | 888.922025 | 1632.304965 |
| TCGA-60-2719 | -698.61797 | | 731.635995 | 33.01802155 |
| TCGA-22-5478 | 195.754319 | | 807.885697 | 1003.640017 |
| TCGA-66-2757 | -142.79585 | | 1088.32627 | 945.5304189 |
| TCGA-18-3406 | 710.965577 | | 1849.94241 | 2560.907989 |
| TCGA-77-A5GB | 306.229566 | | 1433.08769 | 1739.317256 |
| TCGA-21-1070 | 99.376878 | | 2112.61568 | 2211.992559 |
| TCGA-43-A475 | 37.2718336 | | 2578.31645 | 2615.588281 |
| TCGA-85-7698 | 478.772341 | | 1201.68514 | 1680.457476 |
| TCGA-85-7843 | 264.612459 | | 859.212862 | 1123.825322 |
| TCGA-NC-A5HT | -1158.1371 | | -839.04924 | -1997.18638 |
| TCGA-58-8393 | 179.855782 | | 2120.55286 | 2300.408645 |
| TCGA-66-2737 | 1200.41842 | | 1387.49455 | 2587.912965 |
| TCGA-LA-A7SW | -642.24749 | | 395.990957 | -246.256534 |
| TCGA-77-8007 | 779.425057 | | 2102.77899 | 2882.20405 |
| TCGA-85-8354 | -1302.7196 | | 45.586851 | -1257.13279 |
| TCGA-22-5474 | 139.077208 | | 2070.79811 | 2209.875318 |
| TCGA-77-7140 | -312.27422 | | 24.8231901 | -287.451028 |
| TCGA-22-4601 | -704.29646 | | 320.278661 | -384.017795 |
| TCGA-66-2794 | -462.54617 | | 1971.93111 | 1509.384944 |
| TCGA-22-1017 | -507.90859 | | 1272.79051 | 764.881921 |
| TCGA-77-A5GA | -847.264 | | 401.909884 | -445.354121 |
| TCGA-56-7222 | -1147.8243 | | -6.1522227 | -1153.97648 |
| TCGA-85-7699 | 617.316609 | | 2037.74681 | 2655.063419 |
| TCGA-66-2758 | -167.99229 | | 1832.32078 | 1664.328491 |
| TCGA-85-7950 | 586.298019 | | 1597.12432 | 2183.422344 |
| TCGA-39-5034 | 479.401005 | | 919.029593 | 1398.430598 |
| TCGA-85-8052 | -515.93009 | | 528.213099 | 12.28300552 |
| TCGA-43-3394 | -380.64902 | | 313.143242 | -67.505783 |
| TCGA-NK-A5CX | 99.3750432 | | 1266.02667 | 1365.401711 |
| TCGA-39-5035 | -28.739754 | | 1860.51461 | 1831.774858 |
| TCGA-43-6647 | 1360.04264 | | 1748.55081 | 3108.593451 |
| TCGA-18-4086 | 296.724364 | | 1187.83765 | 1484.562009 |
| TCGA-NC-A5HF | -1436.1592 | | -481.78652 | -1917.94568 |
| TCGA-39-5029 | -79.678083 | | 1321.13715 | 1241.459062 |
| TCGA-43-5668 | 336.702344 | | 2216.11287 | 2552.815216 |
| TCGA-22-1012 | -382.60066 | | 869.001815 | 486.4011562 |
| TCGA-34-8454 | 505.041435 | | 2040.63537 | 2545.676803 |
| TCGA-60-2709 | 1327.8019 | | 2367.14757 | 3694.949472 |
| TCGA-85-A512 | -487.27079 | | 299.074186 | -188.196607 |
| TCGA-NC-A5HI | -1395.6636 | | 298.602669 | -1097.06093 |
| TCGA-18-3409 | 1436.9941 | | 1282.10084 | 2719.094942 |
| TCGA-56-8307 | -639.98764 | | 1473.69355 | 833.7059043 |
| TCGA-85-8288 | 495.009628 | | 1008.65874 | 1503.668372 |
| TCGA-18-3410 | -212.42725 | | 1921.03662 | 1708.609371 |
| TCGA-22-1000 | 1553.89366 | | 2350.4736 | 3904.367268 |
| TCGA-66-2778 | -385.99362 | | 1434.37084 | 1048.377224 |
| TCGA-34-5239 | 1099.67353 | | 1908.95373 | 3008.627263 |
| TCGA-34-8455 | -13.080164 | | 600.07902 | 586.9988561 |
| TCGA-66-2765 | 144.699956 | | 1580.73007 | 1725.430024 |
| TCGA-85-8277 | -681.74049 | | 705.327869 | 23.58738351 |
| TCGA-92-8065 | -25.948913 | | 1602.34507 | 1576.396159 |
| TCGA-46-3766 | 1815.37379 | | 2518.81835 | 4334.192141 |
| TCGA-77-8131 | 933.192412 | | 2709.25813 | 3642.450545 |
| TCGA-94-A5I4 | -913.27284 | | 235.413653 | -677.859188 |
| TCGA-18-3407 | 347.025285 | | 868.90249 | 1215.927775 |
| TCGA-77-8139 | -13.528295 | | 622.92636 | 609.3980659 |
| TCGA-21-1078 | -1611.468 | | -267.74488 | -1879.2129 |
| TCGA-18-3411 | 80.3676456 | | 1243.15232 | 1323.519961 |
| TCGA-66-2742 | -29.381717 | | 1090.00039 | 1060.618669 |
| TCGA-56-1622 | -786.68261 | | -234.16862 | -1020.85123 |
| TCGA-77-A5G3 | -646.00562 | | 344.33916 | -301.666461 |
| TCGA-22-5482 | -290.92326 | | 109.24078 | -181.682485 |
| TCGA-NK-A7XE | -1426.5919 | | 397.497678 | -1029.09419 |
| TCGA-22-5489 | 1160.11757 | | 2111.31916 | 3271.436726 |
| TCGA-O2-A52W | -1194.1796 | | 376.572493 | -817.607139 |
| TCGA-52-7812 | -334.75816 | | 1723.46615 | 1388.707988 |
| TCGA-46-6025 | -560.36448 | | 253.311709 | -307.052776 |
| TCGA-58-8391 | -1041.5937 | | 35.9417599 | -1005.65197 |
| TCGA-85-A50M | -40.621969 | | 527.285154 | 486.6631859 |
| TCGA-18-3417 | 886.38537 | | 991.322093 | 1877.707463 |
| TCGA-60-2724 | 515.835567 | | 570.842023 | 1086.67759 |
| TCGA-94-A5I6 | -497.72151 | | 144.269286 | -353.45222 |
| TCGA-43-8116 | -507.01596 | | 1057.93819 | 550.922234 |
| TCGA-34-5241 | 878.847073 | | 1881.25602 | 2760.103096 |
| TCGA-77-7338 | 504.54928 | | 894.572322 | 1399.121602 |
| TCGA-56-7822 | -406.28713 | | 628.420828 | 222.1336951 |
| TCGA-85-A4JC | 233.090587 | | 1243.82666 | 1476.917245 |
| TCGA-66-2792 | -127.73995 | | 437.579418 | 309.8394677 |
| TCGA-98-A53A | -1174.0637 | | 174.839768 | -999.223888 |
| TCGA-22-4594 | 579.803514 | | 1901.03016 | 2480.83367 |
| TCGA-58-8386 | 365.260011 | | 1517.15927 | 1882.419285 |
| TCGA-22-5481 | -613.30463 | | 1310.77837 | 697.4737332 |
| TCGA-66-2754 | -1102.8494 | | -272.73203 | -1375.58141 |
| TCGA-21-1082 | 109.309129 | | 1347.80862 | 1457.117747 |
| TCGA-58-8392 | -1327.8333 | | -151.41954 | -1479.25287 |
| TCGA-60-2713 | 1013.8685 | | 1868.40647 | 2882.274966 |
| TCGA-22-5479 | -251.01607 | | 815.600405 | 564.5843355 |
| TCGA-37-4133 | -227.78902 | | 1217.45967 | 989.6706469 |
| TCGA-63-A5MU | -1499.1226 | | -300.41179 | -1799.53443 |
| TCGA-66-2769 | 888.209044 | | 2030.23917 | 2918.448211 |
| TCGA-LA-A446 | -1208.0458 | | 268.557632 | -939.488169 |
| TCGA-66-2759 | 55.6245105 | | 1383.73157 | 1439.356085 |
| TCGA-22-4609 | 773.649207 | | 2076.20362 | 2849.85283 |
| TCGA-66-2773 | 19.5219533 | | 1602.85672 | 1622.378676 |
| TCGA-66-2755 | 560.726778 | | 2375.96082 | 2936.687601 |
| TCGA-77-8150 | -409.87668 | | 1148.04186 | 738.1651848 |
| TCGA-37-A5EL | -1620.507 | | -26.865822 | -1647.37278 |
| TCGA-77-8009 | -381.56151 | | 223.960168 | -157.601343 |
| TCGA-33-AAS8 | -1651.5095 | | -44.001481 | -1695.51098 |
| TCGA-58-A46N | -1364.8871 | | -284.76614 | -1649.65322 |
| TCGA-33-AASD | -797.53859 | | -34.044424 | -831.583014 |
| TCGA-63-A5MS | 601.758521 | | 1785.24862 | 2387.007142 |
| TCGA-43-A474 | -566.79801 | | -661.0762 | -1227.87421 |
| TCGA-43-6770 | 97.5916157 | | 610.416118 | 708.0077336 |
| TCGA-22-4607 | 168.874281 | | 1024.819 | 1193.693283 |
| TCGA-58-A46K | -863.57273 | | 350.231881 | -513.340845 |
| TCGA-77-7465 | -22.402259 | | 1888.62242 | 1866.220157 |
| TCGA-18-4721 | 668.644336 | | 1876.19619 | 2544.840526 |
| TCGA-85-A4CL | -202.67449 | | 1571.23495 | 1368.560456 |
| TCGA-22-4604 | 1046.90963 | | 1774.45732 | 2821.366951 |
| TCGA-56-7730 | -1723.4726 | | -638.82742 | -2362.30004 |
| TCGA-39-5039 | 624.233162 | | 1073.95429 | 1698.18745 |
| TCGA-J1-A4AH | -1211.4399 | | 77.2968842 | -1134.14302 |
| TCGA-92-8063 | -86.457851 | | 637.179493 | 550.7216418 |
| TCGA-46-3769 | 386.994964 | | 1710.41371 | 2097.408671 |
| TCGA-43-8118 | -3.2828327 | | 1183.45171 | 1180.168882 |
| TCGA-NC-A5HO | -958.12825 | | 622.004867 | -336.123387 |
| TCGA-22-4605 | 650.880752 | | 1347.03068 | 1997.911427 |
| TCGA-60-2714 | -60.146106 | | 1481.36921 | 1421.223102 |
| TCGA-33-6738 | -1074.3428 | | 539.891024 | -534.451763 |
| TCGA-63-A5MV | 368.498696 | | 1450.10279 | 1818.601485 |
| TCGA-85-A4CN | 272.956872 | | 646.777638 | 919.7345101 |
| TCGA-NC-A5HK | -1324.8492 | | 365.605192 | -959.244022 |
| TCGA-43-3920 | 753.571242 | | 1389.79801 | 2143.369254 |
| TCGA-21-1072 | -429.3915 | | 150.155019 | -279.236483 |
| TCGA-98-A53B | -372.9869 | | 1031.76662 | 658.7797226 |
| TCGA-85-8072 | -610.40156 | | 943.855126 | 333.4535681 |
| TCGA-68-A59J | -321.49683 | | 1293.90904 | 972.4122073 |
| TCGA-66-2766 | -371.80195 | | 527.945408 | 156.1434597 |
| TCGA-NC-A5HR | -1313.9285 | | 132.383017 | -1181.5455 |
| TCGA-NC-A5HE | 334.47158 | | 2772.84793 | 3107.319512 |
| TCGA-98-A53D | 1358.75379 | | 2261.50622 | 3620.260005 |
| TCGA-66-2783 | -55.427223 | | 1026.89716 | 971.4699371 |
| TCGA-77-7138 | -839.78901 | | 355.042433 | -484.746581 |
| TCGA-56-8629 | -553.03573 | | 377.59749 | -175.438239 |
| TCGA-79-5596 | -960.88247 | | -150.17993 | -1111.0624 |
| TCGA-63-A5MJ | 310.301145 | | 536.305821 | 846.6069658 |
| TCGA-56-6546 | 350.270848 | | 2032.77225 | 2383.043101 |
| TCGA-51-4081 | -148.78334 | | -428.02746 | -576.8108 |
| TCGA-34-5927 | -828.99653 | | -182.96843 | -1011.96495 |
| TCGA-85-8582 | 984.568718 | | 1052.70494 | 2037.273653 |
| TCGA-77-8154 | -913.17616 | | -436.31723 | -1349.49338 |
| TCGA-77-6844 | -756.31521 | | 616.563214 | -139.751998 |
| TCGA-85-8580 | -1147.9528 | | 647.774497 | -500.178266 |
| TCGA-56-A4ZJ | 1117.71412 | | 1833.4722 | 2951.186322 |
| TCGA-21-1083 | -47.533264 | | 1177.37802 | 1129.844753 |
| TCGA-46-3767 | 1590.07172 | | 2040.43264 | 3630.504357 |
| TCGA-56-5898 | 71.0343607 | | 979.236507 | 1050.270868 |
| TCGA-68-7757 | 436.786031 | | 909.860283 | 1346.646314 |
| TCGA-56-A49D | -276.6908 | | 1693.73368 | 1417.042872 |
| TCGA-18-3412 | -401.26778 | | 735.594785 | 334.3270002 |
| TCGA-37-4132 | -93.332575 | | 1538.75993 | 1445.427355 |
| TCGA-22-5483 | 375.320261 | | 862.877192 | 1238.197453 |
| TCGA-85-A4JB | -371.0695 | | 304.869332 | -66.2001716 |
| TCGA-60-2725 | 803.594389 | | 2266.54808 | 3070.142466 |
| TCGA-77-7463 | -872.45565 | | 564.696756 | -307.75889 |
| TCGA-33-AASJ | -1661.5434 | | -85.113224 | -1746.65662 |
| TCGA-43-7657 | -134.41164 | | -126.93351 | -261.345151 |
| TCGA-22-A5C4 | -905.13312 | | 321.03905 | -584.094071 |
| TCGA-90-7769 | -1075.7235 | | -264.85194 | -1340.57544 |
| TCGA-21-1077 | -33.511998 | | 953.228386 | 919.7163879 |
| TCGA-NC-A5HQ | -946.37213 | | 1206.95375 | 260.581624 |
| TCGA-60-2696 | 27.2318476 | | 1473.77743 | 1501.009279 |
| TCGA-22-4591 | -978.68202 | | 270.6281 | -708.053919 |
| TCGA-77-8143 | -1237.9197 | | 310.512999 | -927.406659 |
| TCGA-NK-A5CR | -1290.688 | | -318.98532 | -1609.67336 |
| TCGA-56-7823 | -1106.5294 | | 51.8482502 | -1054.6812 |
| TCGA-63-A5MN | 1078.49964 | | 2718.08011 | 3796.579754 |
| TCGA-52-7622 | 284.762331 | | 1875.35211 | 2160.114446 |
